# Multiscale profiling of protease activity in cancer

**DOI:** 10.1038/s41467-022-32988-5

**Published:** 2022-10-03

**Authors:** Ava P. Amini, Jesse D. Kirkpatrick, Cathy S. Wang, Alex M. Jaeger, Susan Su, Santiago Naranjo, Qian Zhong, Christina M. Cabana, Tyler Jacks, Sangeeta N. Bhatia

**Affiliations:** 1 Koch Institute for Integrative Cancer Research, Massachusetts Institute of Technology, Cambridge, MA USA; 2 Harvard MIT Division of Health Sciences and Technology, Massachusetts Institute of Technology, Cambridge, MA USA; 3 Program in Biophysics, Harvard University, Boston, MA USA; 4 Microsoft Research New England, Cambridge, MA USA; 5 Department of Biological Engineering, Massachusetts Institute of Technology, Cambridge, MA USA; 6 Department of Mechanical Engineering, Massachusetts Institute of Technology, Cambridge, MA USA; 7 Department of Biology, Massachusetts Institute of Technology, Cambridge, MA USA; 8 Department of Electrical Engineering and Computer Science, Massachusetts Institute of Technology, Cambridge, MA USA; 9 Department of Medicine, Brigham and Women’s Hospital, Boston, MA USA; 10 Broad Institute of Massachusetts Institute of Technology and Harvard, Cambridge, MA USA; 11 Wyss Institute at Harvard University, Boston, MA USA; 12 Howard Hughes Medical Institute, Cambridge, MA USA

**Keywords:** Sensors and probes, Cancer microenvironment, Enzymes, Proteases, Cancer imaging

## Abstract

Diverse processes in cancer are mediated by enzymes, which most proximally exert their function through their activity. High-fidelity methods to profile enzyme activity are therefore critical to understanding and targeting the pathological roles of enzymes in cancer. Here, we present an integrated set of methods for measuring specific protease activities across scales, and deploy these methods to study treatment response in an autochthonous model of *Alk*-mutant lung cancer. We leverage multiplexed nanosensors and machine learning to analyze in vivo protease activity dynamics in lung cancer, identifying significant dysregulation that includes enhanced cleavage of a peptide, S1, which rapidly returns to healthy levels with targeted therapy. Through direct on-tissue localization of protease activity, we pinpoint S1 cleavage to the tumor vasculature. To link protease activity to cellular function, we design a high-throughput method to isolate and characterize proteolytically active cells, uncovering a pro-angiogenic phenotype in S1-cleaving cells. These methods provide a framework for functional, multiscale characterization of protease dysregulation in cancer.

## Abstract (web summary)

The activity of multiple enzymes is dysregulated in cancer, but this cannot always be measured through enzyme expression. Here, the authors develop methods to measure protease activity across the organism, tissue, and single cell scales, and identify protease dysregulation in lung cancer and its response to targeted therapy.

## Introduction

Diverse processes in tumor progression rely on changes in not only the abundance, but also the activity of biomolecules^1,2^. Methods to quantitatively track protein activity within the cellular, tissue, and organismic contexts are therefore critical to advance understanding of cancer biology and to design next-generation precision cancer medicines. While the omics revolution has enabled high-throughput assays of the genome, epigenome, transcriptome, and proteome^3^, it has largely stopped short of queries at the protein activity level—a distinct axis of regulation that is often most proximal to actuated biological function. Although single-cell transcriptomics has enabled characterization of intratumoral heterogeneity^4–6^, and techniques to localize proteins^7,8^ and nucleic acid sequences^9–11^ in situ are starting to enable study of tumors in a spatial context^12^, analogous techniques for single-cell and spatial profiling of enzyme activity have been largely undeveloped.

Methods to analyze enzyme activity at the organism, tissue, and cellular scales could yield new biological insights and open diagnostic and therapeutic avenues in cancer. Recent years have seen a push to develop biosensors that measure biomolecular activity in vivo to generate synthetic signals that can be read out noninvasively^13–19^. For example, in vivo nanoparticle and molecular sensors of enzyme activity have enabled noninvasive detection of cancer^13,14,20–23^, while active glucose uptake has been used for functional imaging of cancer metabolism^24^. However, such in vivo readouts largely treat the body as a black box, sacrificing information on spatial localization within the tumor microenvironment (TME), precluding dissection of phenotypic heterogeneity at the single-cell level, and thus reducing biological interpretation. Therefore, there remains a need for methods capable of generating and unifying molecular activity measurements across biological scales.

**Fig. 1:**
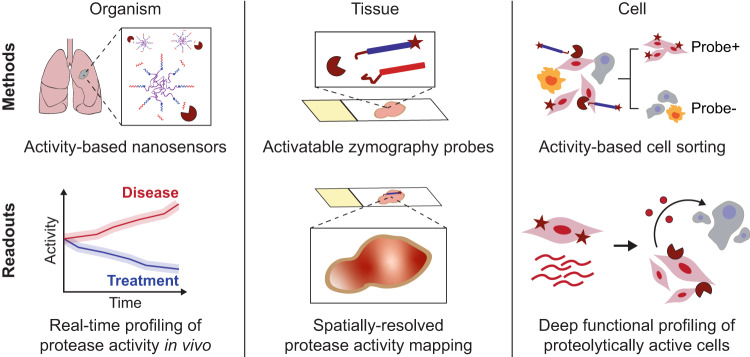
Multiscale profiling of protease activity in cancer. (Top) Methods for profiling protease activity across the organism, tissue, and cellular scales. Noninvasive activity-based nanosensors can be translated into activatable probes for in situ zymography and activity-based cell sorting. (Bottom) Method readouts enable noninvasive, real-time monitoring of in vivo protease activity over tumor progression and treatment response; spatially-resolved activity mapping of the TME; and single-cell isolation and multimodal characterization of proteolytically active cells.

In this work, we present an integrated set of methods to profile protease activity in cancer across the organism, tissue, and cellular scales (Fig. 1). In the in vivo setting, we leverage multiplexed protease-responsive nanosensors together with machine learning to noninvasively detect and longitudinally monitor disease progression in preclinical mouse models. To explore tissue-level organization within the TME, we establish a multiplexed assay for on-tissue spatial localization of protease activity against target peptide substrates. Finally, to link protease activity to other measurement modalities at the cellular scale, we design a single-cell method, termed activity-based cell sorting, that uses peptide probes and flow cytometry to sort individual cells based on their associated enzymatic activity.

We unify these methods into a hierarchical framework (Fig. 1) and apply it to study tumor progression and early drug response in an autochthonous mouse model of *Alk*-mutant non-small-cell lung cancer (NSCLC)^25^. We uncover significant shifts in protease activity that occur after targeted therapy with the ALK inhibitor alectinib. Spatial and single-cell profiling link a treatment-responsive activity signature to pericytes and endothelial cells of the angiogenic tumor vasculature, suggesting dynamic cross-talk between cancer cells and cells of the TME. We envision that these methods to detect protease activity across scales could yield rich functional data about the tumor microenvironment and translate to cancer diagnostics and therapeutics.

## Results

### Profiling protease activity in vivo to monitor tumor progression and treatment response

**Fig. 2:**
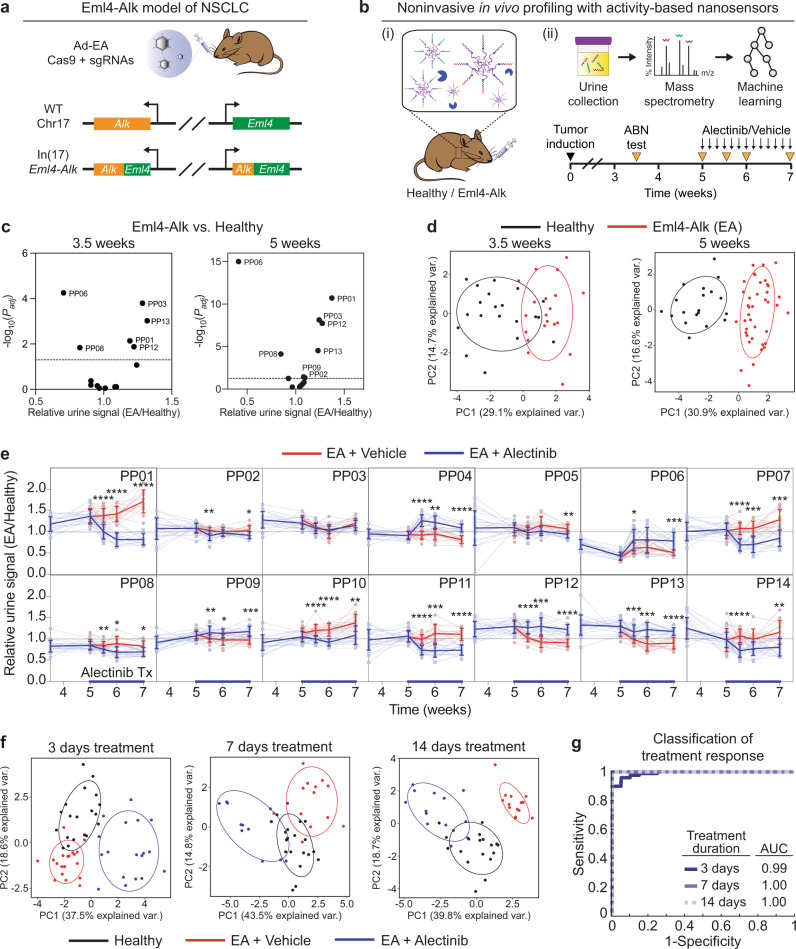
Activity-based nanosensors measure in vivo enzyme activity dynamics over tumor progression and treatment response. **a** Disease induction in the Eml4-Alk model. **b** Schematic of approach. (i) Activity-based nanosensors were administered to Eml4-Alk and healthy mice. (ii) Tumor-bearing Eml4-Alk mice were administered either alectinib or vehicle control and subject to in vivo protease activity profiling (ABN test) over disease progression. **c** Comparison of urinary reporter concentrations in Eml4-Alk (EA) and healthy (Healthy) mice at 3.5 weeks (*n* = 20 per group; left) and 5 weeks (EA, *n* = 40; Healthy, *n* = 19; right) after tumor induction. Significance was calculated by two-sided *t* test with Holm-Sidak correction. Dotted line is at *P*_*adj*_ = 0.05. **d** Principal component analysis (PCA) of urinary reporter output of Eml4-Alk (EA) mice and healthy controls at 3.5 weeks (*n* = 20 per group; left) and 5 weeks (EA, *n* = 40; Healthy, *n* = 19; right) after tumor induction. **e** Healthy control-normalized urinary reporter signal for each of the 14 activity-based nanosensors. Transparent lines show trajectories of each mouse over time; opaque lines are averages over all mice per group. Red lines represent Eml4-Alk mice treated with vehicle (EA + Vehicle; *n* = 20, 19, 13, and 14 for 5, 5.5, 6, and 7 weeks, respectively), and blue lines represent Eml4-Alk mice treated with alectinib (EA + Alectinib; *n* = 20, 19, 12, and 14 for 5, 5.5, 6, and 7 weeks, respectively), with *n* = 20 at 3.5 weeks (mean ± s.d; multiple two-sided *t* tests with Holm-Sidak correction; *P < 0.05, **P < 0.01, ***P < 0.001, ****P < 0.0001). **f** PCA of urinary reporter output of healthy (Healthy), vehicle-treated Eml4-Alk (EA + Vehicle), and alectinib-treated Eml4-Alk (EA + Alectinib) mice at 3, 7, and 14 days post treatment induction. **g** Receiver operating characteristic (ROC) curve showing performance of a random forest classifier in treatment response classification in an independent test cohort at the designated post-treatment time points (*n* = 10 independent trials). Source data are provided in a source data file.

We first sought to establish the ability of our activity-based profiling framework to noninvasively detect and monitor disease over tumor progression and treatment response. We utilized an autochthonous mouse model of ALK^+^ NSCLC as a model system, in which intrapulmonary administration of an adenovirus encoding two guide RNAs and Cas9 resulted in oncogenic rearrangement of the *Eml4* and *Alk* genes (Fig. 2a), leading to the formation of lung tumors that histologically resembled human lung adenocarcinoma^25^. Hereafter, we refer to this *Eml4-Alk* driven model of NSCLC as the Eml4-Alk model. We queried a bulk RNA sequencing (RNA-seq) dataset of Eml4-Alk lungs^26^ and identified several proteases overexpressed in Eml4-Alk mice (Fig. S1). To noninvasively monitor protease activity in the Eml4-Alk model, we engineered a multiplexed panel of activity-based nanosensors that can be selectively activated by dysregulated proteases within the TME to release mass-barcoded peptide reporters that clear into the urine (Fig. 2b)^22^. Critically, these nanosensors (Table S1) use peptide substrates that can be recognized in vitro by a range of metallo-, serine, and aspartic proteases^22^ and that require substrate cleavage for activation and release of the mass-barcoded urinary reporters. Beginning at 3.5 weeks after tumor induction, when tumors are at an early stage of progression^25^, we intratracheally administered the nanosensor panel into Eml4-Alk and healthy control mice, and observed that several nanosensors were differentially cleaved by proteases in the pulmonary microenvironment (Fig. 2c), enabling differentiation of tumor-bearing and healthy mice at an early stage of tumor progression (Fig. 2d).

We then assessed whether activity-based nanosensors could rapidly and quantitatively monitor the dynamics of tumor progression and regression. We treated Eml4-Alk mice with the first-line clinical ALK inhibitor alectinib^27^ and monitored changes in pulmonary protease activity over a two-week treatment course that resulted in rapid and robust tumor regression (Fig. 2b, Fig. S2a, b). Strikingly, we observed that alectinib treatment significantly altered pulmonary protease activity within just 3 days of treatment initiation, with 12 of 14 reporters exhibiting differential enrichment in the urine of vehicle- versus alectinib-treated mice (Fig. 2e). Signal trajectories for each of the individual nanosensors revealed distinct patterns in their dynamics (Fig. 2e). Notably, cleavage of a subset of nanosensors (e.g., PP01, PP07, PP10) increased over time in vehicle-treated mice as tumors progressed but rapidly regressed following alectinib treatment, while the cleavage of other nanosensors (e.g., PP04) transiently increased upon initiation of alectinib treatment and then returned towards baseline levels. Principal component analysis (PCA) revealed that protease activity in vehicle-treated Eml4-Alk mice grew more divergent from healthy controls over the course of tumor progression, whereas that of alectinib-treated mice became more similar to healthy controls (Fig. 2f). As such, a random forest classifier trained on urinary reporter signatures from a subset of Eml4-Alk mice achieved highly accurate classification of therapeutic response to ALK inhibition (Fig. 2g, Table S2).

### Multiplexed spatial localization of protease activity

We next sought to investigate the biological drivers of the observed protease activity dysregulation in Eml4-Alk mice. To this end, we reasoned that tissue-level spatial localization of protease activity against target peptide substrates could facilitate biological interpretation. For instance, understanding where in the tumor microenvironment PP01 is cleaved would point us toward proteolytically active cells that may play important roles in tumorigenesis and thus represent potential diagnostic or therapeutic targets. Because our in vivo nanosensors use peptide cleavage as their mechanism of release and measurement, we translated their substrates into in situ activatable zymography probes (AZPs) that also rely on substrate-specific proteolytic cleavage for activation^28^. Within an AZP, a protease-cleavable substrate links a fluorophore-tagged, positively-charged domain (polyR) with a negatively-charged domain (polyE); this structure remains complexed in the absence of proteolytic activation. When AZPs are applied to fresh-frozen tissue sections in a manner analogous to immunofluorescence staining, substrate cleavage by tissue-resident enzymes liberates the tagged polyR to electrostatically interact with and bind the tissue, enabling localization of protease activity by microscopy.

**Fig. 3:**
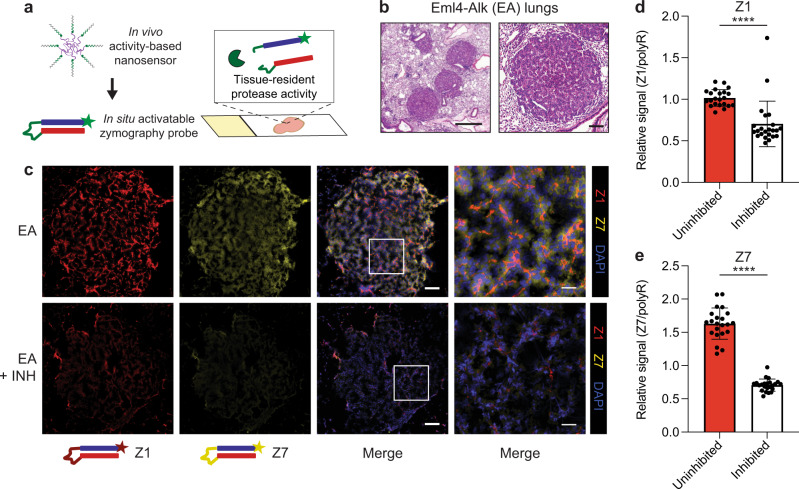
Multiplexed AZPs reveal spatially distinct protease activity patterns. **a** Substrates nominated from in vivo profiling were translated into in situ AZPs to measure and spatially localize tissue-resident enzyme activity in frozen tissue sections. **b** AZPs were applied to fresh-frozen lung tissue sections from Eml4-Alk (EA) tumor-bearing mice. Haematoxylin and eosin (H*&*E) stains of representative Eml4-Alk tumors. Scale bars = 500 μm (left), 100 μm (right). **c** Application of a multiplexed AZP cocktail of Z1 (red) and Z7 (yellow), either uninhibited (EA) or with broad-spectrum protease inhibitors (EA + INH), to Eml4-Alk lung tissue sections. Scale bars = 100 μm. Higher magnification images of boxed regions show localization patterns from multiplexed AZP labeling. Scale bars = 25 μm. Quantification of relative Z1 (**d**) and Z7 (**e**) intensity, normalized to polyR binding control signal on a per-cell basis across Eml4-Alk tumors, either in the absence of protease inhibitors (Uninhibited; *n* = 22 tumors) or in the presence of a broad-spectrum cocktail of protease inhibitors (Inhibited; *n* = 23 tumors) (mean ± s.d.; unpaired two-sided *t* test, ****P < 0.0001). Source data are provided in a source data file.

We thus leveraged AZPs for on-tissue spatial localization of protease activity against target peptide substrates nominated from in vivo profiling (Fig. 2). We selected three nanosensors whose signals tracked with tumor progression and alectinib treatment response (PP01, PP07, PP10; Fig. 2e), and incorporated them into individual AZPs with orthogonal fluorophores (Z1, Z7, Z10, respectively; Fig. 3a). We applied these three AZPs to consecutive sections of lung tissue from Eml4-Alk mice 7 weeks after tumor induction (Fig. 3b) and observed protease-mediated, tumor-specific labeling of Z1 and Z7, but not Z10 (Fig. S3a–c). Intriguingly, we observed that the Z1 staining pattern appeared to follow a spindle-like pattern, distinct from the more diffuse Z7 staining pattern. Thus, we sought to multiplex the three AZPs on a single slide and assess any differences in staining pattern. Once again, we detected protease-mediated, tumor-specific labeling of Z1 and Z7 (Fig. 3c). We also observed fluorescence signal in the Z10 (FITC) channel (Fig. S3d), which we presumed to be due to spectral overlap from the Z7 (TRITC) channel given the results of the single color stain (Fig. S3c). This multiplexed in situ labeling revealed a distinct pattern of spatial localization for Z1 relative to Z7 (Fig. 3c). Qualitatively, while Z7 exhibited broad, diffuse staining throughout Eml4-Alk tumor tissue, Z1 exhibited a prominent spindle-like pattern, suggesting different cells of origin for the proteases cleaving the two probes (Fig. 3c). Tissue labeling of both Z1 and Z7 was significantly abrogated by addition of a cocktail of protease inhibitors (*P* < 0.0001; Fig. 3d, e).

### Delineating protease class- and cell type-specific activity with AZPs

**Fig. 4:**
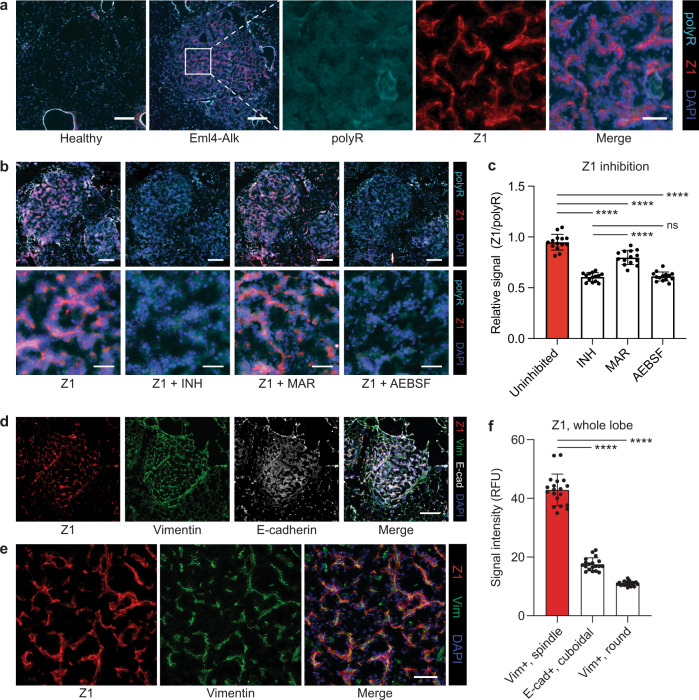
AZPs identify mechanistic class- and cell type-specific protease activity. **a** Staining of lung tissue sections from healthy control and Eml4-Alk mice with Z1 (red), polyR (cyan), and DAPI counterstain (blue). Higher magnification images show staining in a representative Eml4-Alk tumor region. Scale bars = 200 μm, 50 μm (lower and higher magnification, respectively). **b** Application of Z1 to Eml4-Alk lung tissue sections, either alone (Z1) or in the presence of inhibitors: a broad-spectrum cocktail of protease inhibitors (Z1 + INH), the MMP inhibitor marimastat (Z1 + MAR), or the serine protease inhibitor AEBSF (Z1 + AEBSF). Sections were stained with a polyR binding control (cyan) and counterstained with DAPI (blue). Scale bars = 200 μm (top), 50 μm (bottom). **c** Quantification of relative Z1 intensity, normalized to polyR signal, from Eml4-Alk tumors, either in the absence of protease inhibitors (Uninhibited), or in the presence of INH, MAR, or AEBSF (*n* = 14 tumors; mean ± s.d.; unpaired two-sided *t* test, ****P < 0.0001, ^ns^*P* = 0.7127). **d** Application of Z1 (red) to Eml4-Alk lung tissues sections with co-staining for vimentin (green) and E-cadherin (white). Scale bar = 200 μm. **e** Higher-magnification images of a second tumor region, independent of that shown in (**d**), showing Z1 and vimentin stains. Scale bar = 50 μm. **f** Quantification of Z1 staining intensity for per-tumor cell populations, across an entire lung lobe (*n* = 19 tumors, with intensities averaged across all cells in a tumor; mean ± s.d.; paired two-sided *t* test, ****P < 0.0001). Source data are provided in a source data file.

Having demonstrated that orthogonal AZPs could be simultaneously multiplexed, we next endeavored to show that they could be used to identify protease families and cell compartments contributing to their cleavage. Due to its prominent in situ localization pattern and the significant in vivo correlation of PP01 with tumor progression, we nominated Z1 for further investigation and sought to understand the processes driving cleavage of this peptide (“S1” for cleavage motif; Table S3). Whereas healthy lungs exhibited undetectable Z1 staining (Fig. S4a), Eml4-Alk tumors exhibited strong, spindle-like staining that was distinct from the uniform staining pattern of a free polyR binding control (Fig. 4a, Fig. S4b). Furthermore, lungs from alectinib-treated Eml4-Alk mice exhibited fewer regions of Z1 staining (Fig. S4c). To further verify that the Z1 localization pattern truly reflected specific protease expression by the labeled cells, rather than nonspecific labeling (i.e., due to non-uniform distribution of charge), we precleaved Z1 in vitro with recombinant fibroblast activation protein (FAP; Fig. S5) and compared its tissue labeling to that of intact Z1 activated by tissue-resident enzymes (Fig. S6a, b). While intact Z1 maintained its spindle-like spatial pattern (Fig. S6a), precleaved Z1 exhibited diffuse, uniform labeling that mirrored that of a free polyR (Fig. S6b), verifying that probe localization depended on local in situ activation.

To determine class-specific contributions to its cleavage, we applied Z1, whose substrate can be recognized by both matrix metalloproteinases (MMPs) and serine proteases^15,22,29^, to Eml4-Alk lung tissue sections in the absence of protease inhibitors, with a broad-spectrum cocktail of protease inhibitors, with the MMP inhibitor marimastat, or with the serine protease inhibitor 4-(2-aminoethyl)benzenesulfonyl fluoride hydrochloride (AEBSF; Fig. 4b). As expected, incubation with broad-spectrum protease inhibitors significantly abrogated Z1 labeling (*P* < 0.0001; Fig. 4c). Qualitatively, Z1 signal was largely preserved in sections incubated with marimastat (Fig. 4b). While marimastat did reduce Z1 signal, it remained significantly increased relative to the broad-spectrum inhibitor condition (*P* < 0.0001; Fig. 4c). In contrast, incubation with AEBSF completely abrogated Z1 tissue labeling to the level of broad-spectrum inhibition (*P* < 0.0001 uninhibited vs. AEBSF, *P* = 0.7127 broad-spectrum inhibition vs. AEBSF; Fig. 4c), suggesting that serine proteases are primarily responsible for cleaving Z1 in Eml4-Alk tumors.

Though Eml4-Alk tumors are adenocarcinomas and thus consist primarily of epithelial cells, the distinct spindle-like labeling pattern of Z1 raised the possibility that Z1 might be cleaved by proteases expressed by non-epithelial cells of the TME. To this end, we applied Z1 to Eml4-Alk lung tissue sections and simultaneously stained for both E-cadherin, an epithelial cell marker, and vimentin, the intermediate filament of mesenchymal cells (Fig. 4d, e, Fig. S7a, b). We observed an overlap between Z1 and vimentin in Eml4-Alk tissue (Fig. 4e), confirmed by staining for the aforementioned two markers alone (Fig. S8a). In contrast, Eml4-Alk tumor regions exhibited distinct localization of Z1 relative to epithelial cells marked by E-cadherin (Fig. S8b), while there was diminished Z1 labeling in healthy lungs altogether (Fig. S8c, d). Quantitatively, vimentin-positive, spindle-like cells exhibited increased Z1 intensity relative to E-cadherin-positive cells or vimentin-positive, rounded cells (likely tumor-associated macrophages) (*P* < 0.0001, see Methods for details; Fig. 4f). These results suggest that vimentin-positive, spindle-like cells are associated with the serine protease activity cleaving Z1 and, more broadly, that AZPs can delineate class-specific and cell type-associated activity patterns.

### Multimodal spatial profiling to functionally query the TME

**Fig. 5:**
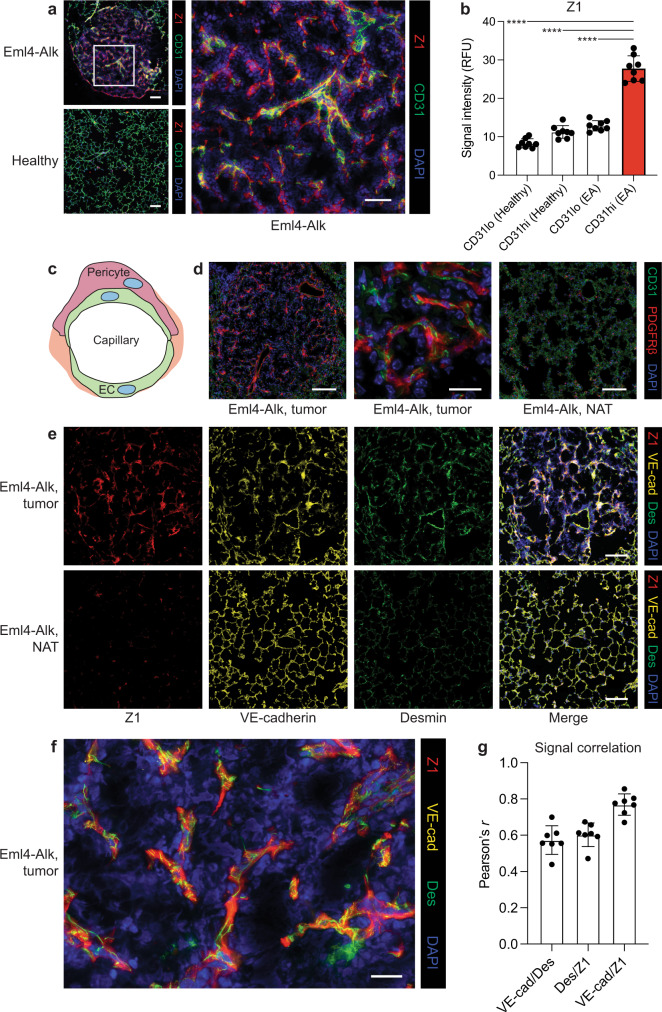
Z1 localizes to pericyte-invested vasculature in Eml4-Alk tumors. **a** Application of Z1 (red) to Eml4-Alk and healthy lung tissue sections with co-staining for CD31 (green). Scale bars = 100 μm, 50 μm (lower and higher magnification, respectively). **b** Quantification of Z1 staining intensity in CD31-low (CD31lo) and CD31-high (CD31hi) cells (*n* = 8 regions per condition; mean ± s.d.; one-way ANOVA with Tukey correction for multiple comparisons, ****P < 0.0001). **c** Capillary vessels are lined by endothelial cells (EC); pericytes support and wrap around endothelial cells. **d** Staining for endothelial cells (CD31; green) and pericytes (PDGFR*β*; red) in formalin-fixed, paraffin-embedded Eml4-Alk lung tissue sections, with images from representative tumor (left, middle) and normal adjacent tissue (NAT; right) regions shown. Scale bars = 100 μm, 20 μm (lower and higher magnification, respectively). **e** Application of Z1 (red) to Eml4-Alk lung tissues with co-staining for VE-cadherin (VE-cad; yellow) and desmin (Des; green). Scale bars = 100 μm. **f** Higher magnification image of representative Eml4-Alk tumor region showing localization of Z1, VE-cadherin, and desmin. Scale bar = 20 μm. **g** Pearson's correlation of pixel-wise signal intensities for each pairwise combination of Z1, VE-cadherin, and desmin (*n* = 7 tumors; mean ± s.d.). Source data are provided in a source data file.

Next, we sought to further investigate the cell type(s) responsible for Z1 cleavage, as their protease activity suggested aberration and potential contribution to tumor progression. The distinct spatial pattern of Z1 staining led us to hypothesize that this probe could be labeling cells of the tumor vasculature, rather than cells of immune or other mesenchymal compartments. We thus applied Z1 to Eml4-Alk and healthy lungs and co-stained for the endothelial cell marker CD31 (PECAM-1; Fig. S9a). Qualitatively, while both Eml4-Alk and healthy lungs exhibited an abundance of endothelial cells as evidenced by CD31-positivity, Z1 labeling was enriched in Eml4-Alk tumors relative to healthy lungs and tended to colocalize with CD31-positive cells (Fig. 5a). Cell-by-cell quantification of Z1 and CD31 staining intensities across entire lung tissue sections identified a strong positive correlation in Eml4-Alk tissue (*R*^2^ = 0.67; Fig. S9b). Indeed, Z1 staining was significantly increased in CD31-high cells in Eml4-Alk lung tissue sections relative to CD31-high cells in healthy lungs, as well as relative to CD31-low cells in both Eml4-Alk and healthy tissue (*P* < 0.0001; Fig. 5b), suggesting specific activity associated with the Eml4-Alk tumor endothelium. Furthermore, immunostaining for VE-cadherin, a strictly endothelial-specific adhesion molecule^30^, revealed a spindle-like pattern of expression within Eml4-Alk tumors that mimicked Z1 staining (Fig. S10).

In addition to endothelial cells, the vasculature also contains contractile vascular smooth muscle cells that line the vessel walls. Capillaries and microvessels, such as those within the lungs, contain a mural, periendothelial mesenchymal cell population known as pericytes (Fig. 5c), which help regulate vascular function and can be actively recruited into the vasculature during angiogenesis^1,31,32^. Eml4-Alk tumors stained positively for *α*-smooth muscle actin (*α*SMA; Fig. S11a), a canonical vascular smooth muscle cell marker that can be expressed by tumor pericytes but is often absent in quiescent pericytes in normal tissues^31,32^. Indeed, normal adjacent tissue (NAT) showed reduced *α*SMA expression (Fig. S11a). To further corroborate the likely presence of pericytes within the tumor vasculature, we stained Eml4-Alk and healthy lungs for CD31 and a second pericyte marker, the muscular intermediate filament desmin^31^, and observed desmin-positive cells surrounding CD31-positive endothelial cells within Eml4-Alk tumors but not in NAT (Fig. S11b) nor healthy tissue (Fig. S11d). Finally, we stained Eml4-Alk and healthy lung tissue sections for the pericyte-associated marker PDGFR*β*. The PDGF-B/PDGFR*β* signaling pathway is a key axis of interaction between endothelial cells and pericytes, wherein PDGF-B released from angiogenic endothelial cells binds to PDGFR*β* on the surface of pericytes, facilitating their recruitment^31,33^. Eml4-Alk tumors stained positively for both CD31 and PDGFR*β*, while NAT from Eml4-Alk lungs (Fig. 5d, Fig. S11c) and healthy tissue from healthy lungs (Fig. S11e) did not express PDGFR*β* despite abundant CD31 expression. Within the tumor vasculature specifically, PDGFR*β*-positive cells wrapped around CD31-positive cells, consistent with the expected localization and function of pericytes (Fig. 5d, Fig. S11c).

To assess its localization with respect to cells of the tumor vasculature, we applied Z1 to Eml4-Alk lung tissue sections with concurrent staining for both the endothelial marker VE-cadherin and the pericyte marker desmin. We observed robust Z1 labeling together with VE-cadherin and desmin expression within Eml4-Alk tumors (Fig. 5e). However, NAT displayed decreased Z1 and desmin staining despite maintaining VE-cadherin positivity. Closer inspection of Z1 labeling within Eml4-Alk tumors revealed an association between all three stains (Fig. 5f). Colocalization analysis demonstrated a correlation between desmin and VE-cadherin staining, consistent with the close proximity of both cell types within capillaries, and additionally showed that both desmin and VE-cadherin correlated with Z1 labeling (Fig. 5g). Through coupling of AZPs with other spatial measurements, these results suggest that the sensor S1 reads out specific functional (i.e., serine protease activity) and compositional (i.e., increased pericyte coverage) changes within the Eml4-Alk TME.

### Complementing protease activity measurements with single-cell transcriptomics to characterize the Eml4-Alk TME

**Fig. 6:**
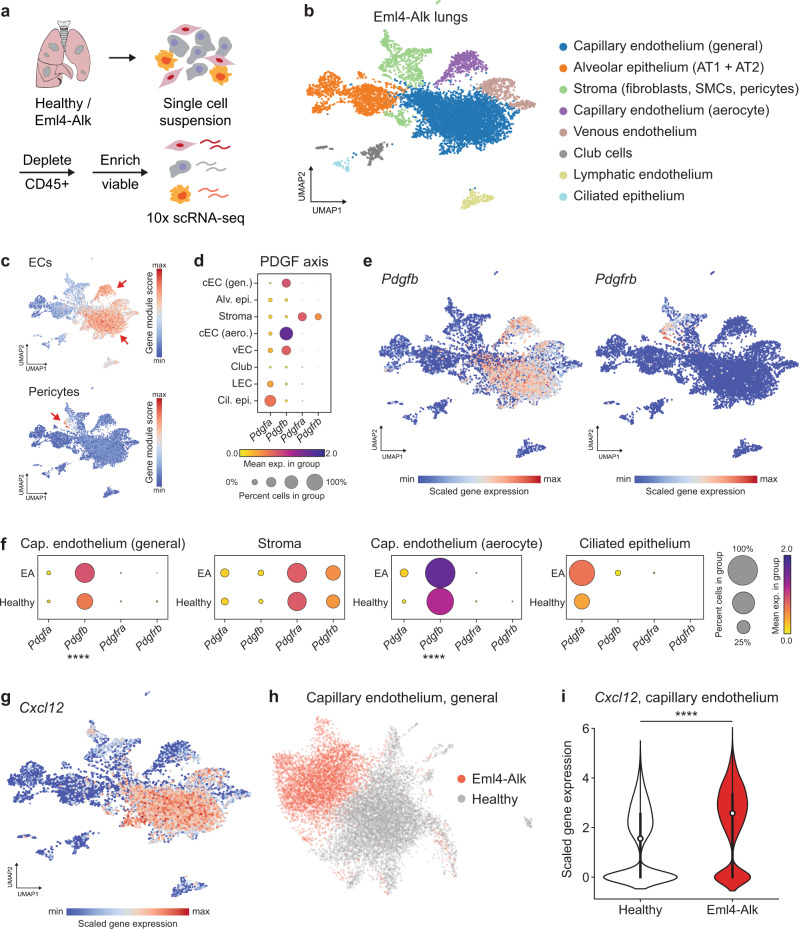
Single-cell transcriptomics of Eml4-Alk tumors reveals dysregulation of the PDGF-CXCL12 signaling axis involved in pericyte recruitment to the angiogenic vasculature. **a** Schematic of workflow. **b** UMAP of scRNA-seq dataset from Eml4-Alk lungs (pooled sample from *N* = 3 mice; *n* = 8127 cells). Cell types were inferred based on expression of canonical marker genes (Table S4). AT1 alveolar type 1, AT2 alveolar type 2, SMC smooth muscle cell. **c** Feature plots of gene expression module scores for endothelial cell (EC) and pericyte marker genes mapped onto the UMAP of cells from Eml4-Alk lungs. **d** Relative expression levels of PDGF and PDGFR genes across cell types in Eml4-Alk lungs. Individual dots represent mean expression values across all cells in a cluster, are colored by expression level, and are sized by the percentage of cells in the cluster expressing that gene. Cluster abbreviations refer to the cell types annotated in (**b**). **e** Expression levels of *Pdgfb* and *Pdgfrb* against the UMAP of cells from Eml4-Alk lungs. **f** Relative expression levels of PDGF and PDGFR genes in cells from Eml4-Alk (EA) and healthy (Healthy) lungs withinin each of the capillary endothelium (general), stromal, capillary endothelium (aerocyte), and ciliated epithelium populations (Wilcoxon rank-sum two-sided test with Benjamini-Hochberg correction, ****P_*adj*_ < 0.0001). **g** Expression levels of *Cxcl12* against the UMAP of cells from Eml4-Alk lungs. **h** UMAP of integrated dataset of cells from capillary endothelium (general) populations in Eml4-Alk and healthy lungs (pooled samples of *N* = 3 mice per condition; *n* = 12,183 cells total with n_*EA*_ = 4766 and *n*_*H*_ = 7447 cells from Eml4-Alk and healthy lungs, respectively). **i** *Cxcl12* expression in capillary endothelial cells from Eml4-Alk and healthy lungs (pooled samples of *N* = 3 mice per condition; *n* = 12,183 cells total with *n*_*EA*_ = 4766 and *n*_*H*_ = 7447 cells from Eml4-Alk and healthy lungs, respectively; Wilcoxon rank-sum two-sided test with Benjamini-Hochberg correction, ${\log }_{2}\,{{\mbox{FC}}}\,=1.453{,}{*\ast*\ast }{P}_{adj}\, < \,0.0001$; white dot, median; thick bar, interquartile range; thin line, minimum to maximum). Source data are provided on GEO under accession number GSE191079.

We next sought to characterize further the phenotypes of the identified S1-associated, tumor vasculature cell populations and to understand potential mechanisms for the dysregulation of these cells. To complement our in vivo and in situ activity measurements, we performed single-cell RNA sequencing (scRNA-seq) to obtain an unbiased view of the cellular and transcriptomic landscape of Eml4-Alk lungs (Fig. 6a, Figs. S12a–d, S13). Graph-based clustering of uniform manifold approximation and projection (UMAP) captured the transcriptomic landscape of Eml4-Alk lungs (Fig. S14a, b), where we annotated eight significant groups of cell types based on expression of previously reported marker genes^34,35^ (Fig. 6b, Table S4). Given that S1 cleavage in situ localized to cells of the tumor vasculature, we defined marker gene modules for both endothelial and pericyte populations and computed their expression scores across all cells in Eml4-Alk lungs (Fig. 6c). The identified population of endothelial cells expressed several markers within a module of 28 genes canonically associated with angiogenesis (Figs. S15a, b, S16).

Spatial profiling had indicated the presence of cells positive for each of *α*SMA, desmin, and PDGFR*β* within Eml4-Alk tumors but not within NAT or healthy lung tissue (Fig. S11, Fig. 5), raising the question of whether pericytes were specifically recruited into the TME. We first generated scRNA-seq data from healthy mouse lungs (Fig. S17a–d). Marker gene analysis revealed a small population of pericytes within the larger Eml4-Alk stromal cluster (Fig. 6c, Fig. S18a, b), as well as a small population of cells exhibiting this signature in healthy lungs (Fig. S18c, d), in line with pericyte identification reported in previous transcriptomic cell atlas studies in human^35^ and mouse^34^ lungs. PDGF signaling has been shown to facilitate recruitment of pericytes into the tumor vasculature as a means to stabilize vessels and promote the establishment of an angiogenic, reactive TME^36,37^. We therefore queried expression of both PDGF ligands (*Pdgfa*, *Pdgfb*) and receptors (*Pdgfra*, *Pdgfrb*) in the Eml4-Alk scRNA-seq dataset and found that expression of *Pdgfra* and *Pdgfrb* was exclusive to the stromal cluster (Fig. 6d). This analysis also revealed robust and specific expression of *Pdgfb* in endothelial cell populations (Fig. 6d). Visualization via UMAP corroborated that expression levels of *Pdgfb* and *Pdgfrb* matched the distributions of endothelial cell and pericyte populations, respectively (Fig. 6e).

These results raised the possibility of paracrine PDGF signaling between endothelial cells and PDGFR-positive stromal cells in Eml4-Alk tumors. To investigate whether this axis was transcriptionally dysregulated, we conducted an integrative analysis of scRNA-seq data from Eml4-Alk and healthy lungs (Fig. S19a–d). Differential gene expression analysis across this integrated dataset showed that *Pdgfb* was overexpressed in cells from both capillary endothelial cell compartments in the TME relative to healthy lungs (*P*_*adj*_ < 0.0001; Fig. 6f, Table S5). However, expression of the PDGF receptors *Pdgfra* and *Pdgfrb* remained consistent between the total stromal populations from both conditions (Fig. 6f, Table S5).

These observations motivated the hypothesis that altered ligand expression by endothelial cells in the Eml4-Alk TME could be implicated in the association of pericytes to the tumor vasculature. In addition to *Pdgfb*, the chemokine *Cxcl12*, shown to play functional roles in angiogenesis and vascular recruitment of stromal cells^38^, was robustly expressed in endothelial cells from Eml4-Alk lungs (Fig. 6g). Endothelial cells from Eml4-Alk and healthy lungs exhibited differential transcriptional landscapes (Fig. 6h), with *Cxcl12* expression significantly increased in endothelial cells from Eml4-Alk lungs relative to those from healthy controls (${\log }_{2}\,{{\mbox{FC}}}\,=1.453,\;{P}_{adj}\, < \,0.0001$; Fig. 6i, Table S5). Intriguingly, previous reports have shown that overexpression of PDGF-B can increase tumor pericyte content via induction of CXCL12 expression by endothelial cells within the TME^38^.

Finally, the rapid and profound reduction in PP01 signal (in vivo) and Z1 staining (in situ) after treatment with alectinib, which theoretically should only induce apoptosis in ALK+ cancer cells, led us to investigate the role that *Alk*-mutant cancer cells themselves play in regulating the angiogenic TME. To this end, we established tumor organoids in vitro by inducing Eml4-Alk fusions in alveolar type 2 (AT2) organoids via CRISPR-Cas9^39^. Transcriptomic analysis of Eml4-Alk-mutant organoids revealed enrichment of genes associated with angiogenesis, including *Pdgfb* (Fig. S20a–c), suggesting that *Alk*-mutant cancer cells themselves may contribute directly to endothelial cell and pericyte recruitment. These results also suggest a potential mechanism by which alectinib treatment may indirectly impact the tumor vasculature and its associated protease activity.

### Activity-based cell sorting enables multimodal phenotypic characterization of Eml4-Alk lung cancer

**Fig. 7:**
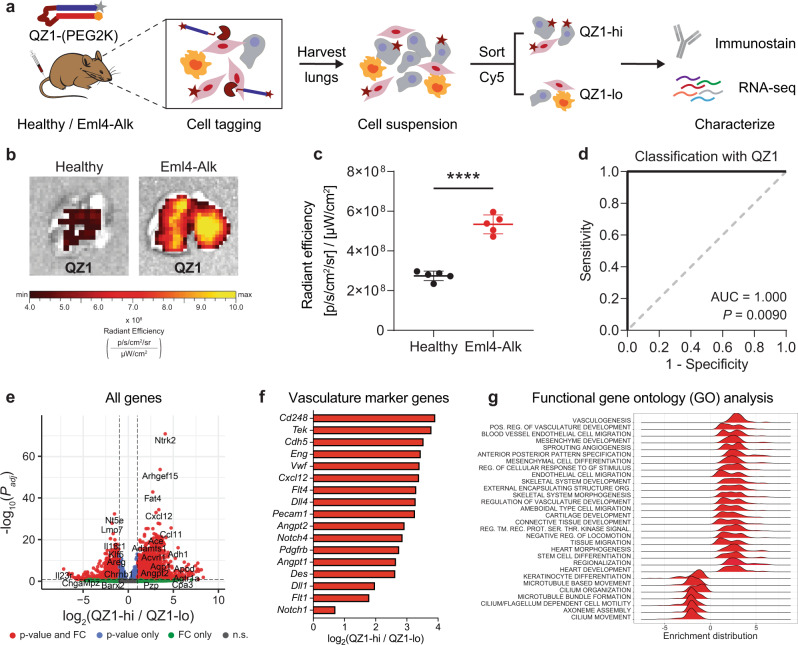
Activity-based cell sorting enables multimodal phenotypic characterization of proteolytically active cells of the tumor microenvironment. **a** The quenched probe QZ1-(PEG2K), consisting of a Cy5-tagged polyR (gray star + blue rectangle) and quencher-tagged polyE (orange hexagon + red rectangle), was administered intravenously into age-matched healthy and Eml4-Alk mice. Lungs were excised, imaged, and dissociated into single cell suspension. Cells were sorted on Cy5 fluorescence and then characterized via immunostaining and bulk RNA-seq. **b** Images of representative lungs from healthy and Eml4-Alk mice 2 h after QZ1-(PEG2K) administration. **c** Quantification of epifluorescence radiant efficiency from experiment in (b) (*n* = 5 mice per group; mean ± s.d.; unpaired two-sided *t* test, ****P < 0.0001). **d** ROC curve showing performance of QZ1-(PEG2K) signal in discriminating healthy from Eml4-Alk lung explants (AUC = 1.000, 95% confidence interval 1.000–1.000; *P* = 0.0090 from random classifier shown in dashed line). **e** Differential expression analysis of RNA-seq data from sorted QZ1-hi and QZ1-lo cells. Each point represents one gene and is colored according to the significance level for that gene. Significance was calculated by Wald test followed by adjustment for multiple hypotheses using the Benjamini-Hochberg correction. Dotted line is at *P*_*adj*_ = 0.05. **f** Identification of vasculature marker genes significantly overexpressed (*P*_*adj*_ < 0.05) in the QZ1-hi population. **g** Enrichment distributions for gene ontology modules up- and downregulated in QZ1-hi relative to QZ1-lo cells. Source data are provided on GEO under accession number GSE191079 and in a source data file.

Our results thus far suggested that alectinib, a therapy targeted toward ALK+ cancer cells, induced rapid and dramatic changes in the proteolytic activity of presumably ALK- vascular cells within the TME. Follow-up transcriptomic profiling unearthed a potential mechanism of communication, mediated by PDGF and CXCL12, between ALK+ cancer cells, endothelial cells, and pericytes. However, as the protease profiling and transcriptomic methods were decoupled, it is impossible to prove that the cells analyzed in the transcriptomic experiments were equivalent to the proteolytically active cells identified in our in situ experiments. We therefore sought to establish a method to isolate individual cells on the basis of their protease activity. We hypothesized that AZPs containing fluorophore-quencher pairs could function as activatable cellular tags in vivo to label cells with membrane-bound or proximal protease activity, such that tagged cells could be subsequently sorted via flow cytometry (Fig. 7a). In this design, following systemic administration, degradation of the protease-cleavable linker activates fluorescence and liberates the fluorophore-tagged polyR such that it can bind and tag nearby cells, functioning analogously to a cell penetrating peptide^28,40,41^. Thus, we reasoned that probe labeling after proteolytic activation (e.g., by cell-surface or proximal proteases) would facilitate isolation of tagged cells via fluorescence-activated cell sorting (FACS) and enable downstream phenotypic characterization (Fig. 7a).

We applied this activity-based cell sorting assay to directly isolate and then phenotypically characterize the Eml4-Alk cell compartment associated with S1 cleavage (Fig. 7a). We designed a fluorescent quenched probe, QZ1, that incorporated S1 as a protease-cleavable linker. Cy5-labeled QZ1 was PEGylated to improve stability and drive tissue accumulation^28,40^, and administered intravenously into age-matched Eml4-Alk and healthy mice. Eml4-Alk mice were assessed at 12 weeks post tumor induction, at which point the lungs contain multiple lung adenocarcinoma lesions^25^. Two hours post-injection, significantly increased Cy5 fluorescence was found in explanted Eml4-Alk lungs relative to healthy lungs (*P* < 0.0001; Fig. 7b, c), enabling perfect discrimination of Eml4-Alk and healthy mice (AUC = 1.000; Fig. 7d).

Following imaging, single-cell suspensions were prepared from dissociated Eml4-Alk lungs, and fluorescence-activated cell sorting (FACS) was used to sort all live, non-hematopoietic nucleated cells by QZ1 signal, demonstrating the feasibility of the activity-based cell sorting method (Fig. 7a, Fig. S21a–b). We also performed co-immunostaining for markers associated with mesenchymal cell lineages. Intriguingly, whereas Eml4-Alk lungs exhibited increased QZ1 staining across cells positive for the mesenchymal cell markers (Fig. S22a–c), in a separate experiment healthy lungs exhibited a more variable pattern (Fig. S23a–c). Bulk RNA-seq on sorted QZ1-high (QZ1-hi) and QZ1-low (QZ1-lo) populations from Eml4-Alk lungs was used to characterize gene expression differences between the two compartments (Fig. 7e, Fig. S24). Several canonical markers of endothelial cells (*Cdh5*, *Eng*, *Vwf*, *Pecam1*), pericytes (*Cd248*, *Pdgfrb*, *Des*), as well as vascularization and angiogenesis were among the most upregulated genes in the QZ1-hi population (Fig. 7f). Gene set enrichment analysis corroborated that the dominant cell types associated with QZ1 labeling were endothelial and mesenchymal cell types, including pericytes, while gene sets associated with epithelial cells were significantly downregulated (Fig. S25a). *Cxcl12* and *Pdgfrb* were overexpressed in the QZ1-hi compartment, as were markers of additional angiogenesis signaling axes, including the VEGF (*Flt1/4*), Notch (*Notch1/4*, *Dll1/4*), and Tie (*Tek*, *Angpt1/2*) pathways (Fig. 7f). Indeed, the QZ1-hi compartment was significantly enriched for functional modules associated with vasculogenesis, vascular development, endothelial cell migration, and mesenchymal recruitment (Fig. 7g, Fig. S25b). These results provide further evidence that S1 is cleaved by proteases associated with the angiogenic tumor vasculature. More broadly, they validate that activity-based cell sorting can be used to isolate cells on the basis of their protease activity, enabling deep phenotypic characterization.

## Discussion

Our results establish a workflow for profiling protease activity across multiple scales–at the organism, tissue, and cellular levels–and demonstrate the utility of our methods for noninvasive monitoring and functional characterization of tumor progression and treatment response, showcased in the context of *Alk*-mutant lung cancer treated with targeted therapy (Fig. 1). We first demonstrated that multiplexed panels of protease-responsive nanosensors can quantitatively track disease dynamics in vivo to yield activity-based biomarkers of tumor progression and response to targeted therapy. We directly translated substrates nominated from in vivo profiling into in situ protease activity probes (AZPs). We thus identified a tumor-specific serine protease activity signal that increased with tumor progression, rapidly decayed after therapy, and localized specifically to the pericyte-invested tumor vasculature. We complemented our activity measurements with single-cell transcriptomic analysis, which identified overexpression of paracrine signaling factors in endothelial cells from tumor-bearing lungs and suggested a possible mechanism for endothelial cell-pericyte cross-talk within the TME. Finally, we designed a high-throughput method to isolate cells based on their protease activity and leveraged it to discover a population of proteolytically active, vasculature-associated cells harboring pro-angiogenic transcriptional programs. Together, these methods revealed that the functionally aberrant tumor vasculature rapidly responds to targeted inhibition of oncogenic signaling in cancer cells and demonstrated that protease activity serves as an informative proxy for this process.

Our work establishes a multiplexed in situ activity assay that enables direct on-tissue comparison of spatial localization patterns of distinct proteases. However, by relying on fluorescence readouts, the current AZP design is limited in its multiplexing capacity. Expanded multiplexing capacity, for example through epitope tagging or DNA barcoding, could enable high-throughput screens on murine or human tissue to discover protease activity sensors with desired properties, such as colocalization with specific cell types. We also demonstrate that AZPs can be used to delineate protease class-specific activity signatures through targeted inhibitor ablations. Given that our results indicate that serine proteases cleave S1 in the Eml4-Alk model (Fig. 4b, c), additional molecular profiling will be necessary to identify which protease(s) are responsible. The angiogenesis-associated proteases *Plat* (tPA) and *Dpp4* (DPP4), as well as the membrane serine protease *Fap* (FAP or seprase), which can be selectively expressed by tumor pericytes^42,43^, were amongst the serine proteases overexpressed in the QZ1-hi population (Fig. S24). In vitro screening, targeted small molecule inhibition, or specific knockout of individual enzyme targets could help identify the serine protease(s) that cleave S1 in the Eml4-Alk model. Parallel functional studies, in particular in tumor-derived organoids or vascularized co-culture systems, may help determine the specific contributions of candidate serine proteases to vascular remodeling and angiogenesis in ALK+ lung cancer.

This work establishes AZPs as an activity-based cellular tag for sorting individual cells based on endogenous protease activity. Administration of AZPs in vivo, followed by tissue dissociation and FACS, enabled isolation of cells exhibiting a specific pattern of protease activity. By coupling this assay to immunostaining and RNA-seq, we demonstrate that activity-based cell sorting can enable multimodal characterization across the activity, protein, and gene expression levels. Probes similar in concept to AZPs could extend activity-based cell sorting to other classes of enzymes. In addition, integrating activity-based cell sorting with large-scale omics measurements and machine learning could inspire single-cell multiomics that ends at the level of actuated biological function. We envision that the ability to sort cells by enzymatic activity could yield insights into enzymatic dysregulation in disease, enable multimodal approaches to characterize biological systems, and inform diagnostic and therapeutic interventions.

Our results demonstrate that protease activity directly complements measurements of protein and transcript abundance, and that this multimodal profiling enables discovery-based functional characterization of the TME. By applying our activity-based profiling methods to the Eml4-Alk model of NSCLC, we discovered aberrant serine protease activity that is specific to the tumor vasculature and rapidly responds to inhibition of an adjacent cancer-cell specific pathway. Through a combination of spatial profiling and scRNA-seq analysis, we found evidence suggestive of increased pericyte coverage within the Eml4-Alk tumor vasculature, potentially mediated by altered paracrine signaling via PDGF and CXCL12. PDGF-expressing endothelial cells can produce CXCL12, a chemokine shown to facilitate recruitment of stromal cells and to promote angiogenesis. This represents one altered function of the Eml4-Alk vasculature, whose dysregulation and angiogenic phenotype can be read out by altered protease activity measured by the sensor S1. Additional investigation into CXCL12 induction and its downstream effects will help determine whether or not chemokine production depends causally on the protease activity, or if it is a parallel altered function of the dysregulated tumor vasculature.

Though mechanistic experiments will be necessary to ascertain whether pericytes are actively recruited into the TME, our findings raise the possibility that S1 cleavage, which is elevated within Eml4-Alk tumors and localizes specifically to the vasculature, could be a result of the coordinated action of intratumoral pericytes and endothelial cells associated with neoangiogenic vessels. Necessary future work to establish tractable ex vivo models, such as vascularized Eml4-Alk tumor organoids or co-culture systems, will in turn enable such functional studies to identify and validate mechanistic targets. Our finding that the functionally aberrant tumor vasculature rapidly responds to targeted therapy motivates exploration of whether anti-angiogenic drugs, which have been clinically approved in combination with cytotoxic chemotherapy or immunotherapy^44–46^, could have additive benefits when combined with molecularly targeted therapeutics like alectinib. Our study in the Eml4-Alk model serves as an example for how our activity profiling methods can be leveraged to spawn and advance hypotheses about the complex crosstalk between cancer and non-cancer cells, though complementary mechanistic and functional work is necessary to validate these hypotheses and establish causal mechanisms.

Finally, the activity-based profiling methods presented here could have utility in precision medicine applications. Precision cancer medicine requires granular information that cannot be accessed by traditional noninvasive imaging approaches, necessitating serial biopsies that carry significant risks and sample only a small fraction of the disease site. The ability to gain high-dimensional biological insight into a disease state with a completely noninvasive test would present an advance towards functional precision medicine^2^. Here, we establish the capacity of noninvasive, multiplexed protease activity nanosensors to query the function and activity of specific intratumoral cell subsets over the course of tumor progression and in response to therapy. Given the modularity of this approach, high-throughput screening^47–49^ and generative machine learning^50^ methods could optimize activity sensors to target orthogonal axes of cancer biology. For instance, activity sensors that detect angiogenesis could be administered in combination with probe sets that read out immune invasion or metastasis risk. As a complement to this noninvasive test, a targeted panel of in situ AZPs could be used to molecularly profile individual patient biopsies for indication of signaling pathways or processes active in a patient’s specific tumor. Future work to validate these technologies in humans, through clinical trials or ex vivo assays on human tissue, will be necessary to assess the robustness and clinical utility of these activity-based methods for precision oncology. With thorough validation and clinical testing, protease activity sensors could empower patients and physicians with real-time, high-quality information to personalize treatment decisions, such as rapid prediction of immunotherapy efficacy, surveillance for recurrence after targeted therapy, or discrimination of aggressive versus indolent disease.

In summary, we present an integrated suite of protease activity-profiling methods that form a direct link between noninvasive enzyme sensors, high-resolution spatial profiling, and high-throughput, single-cell analytical methods like flow cytometry and RNA-seq. The modular methods described here can be readily generalized to other cancer types and hold promise for both fundamental biological investigation and translational research. We envision that these methods for profiling protease activity will help facilitate functional characterization of cancer for medical and discovery applications alike.

## Methods

### Eml4-Alk mouse model of non-small-cell lung cancer

All animal studies were approved by the Massachusetts Institute of Technology (MIT) Committee on Animal Care (protocol 0420-023-23) and were conducted in compliance with institutional and national policies. Reporting was in compliance with Animal Research: Reporting In Vivo Experiments (ARRIVE) guidelines. Tumors were initiated in 6-10 week old female C57BL/6J mice (Jackson Labs) by intratracheal administration of 50 *μ*L adenovirus expressing the Ad-EA vector (VQAd Cas9 ALK EML4 072415; Viraquest; 1.5*10^8^ PFU in Opti-MEM with 10 mM CaCl_2_)^25,51^. Throughout the manuscript, these mice are referred to as “Eml4-Alk” mice. Due to the autochthonous nature of the tumor model and a lack of available means to reliably assess lung tumor volume noninvasively, a maximal tumor volume was not directly established for the experiments in this study. Instead, mice were closely monitored to ensure that they did not exhibit signs of morbidity, including weight loss, poor body condition, or labored breathing. Criteria for euthanasia, as dictated by the MIT Committee on Animal Care, was body weight loss of greater than 10%, significant dyspnea, or poor body condition. Animals were monitored daily throughout all studies, and the criteria for euthanasia were not met. Healthy control cohorts consisted of age- and sex-matched mice (i.e., female C57BL/6J, Jackson Labs) that did not undergo intratracheal administration of Ad-EA adenovirus.

### Alectinib treatment

Eml4-Alk mice were randomized to receive either control vehicle or alectinib (MedChemExpress), at 20 mg/kg prepared directly in drug vehicle, daily by oral gavage for 14 consecutive days. Drug vehicle consisted of: 10% (v/v) dimethylsulfoxide (DMSO; Sigma Aldrich), 10% (v/v) Cremophor EL (Sigma Aldrich), 15% (v/v) poly(ethylene glycol)-400 (PEG400; Sigma Aldrich), 15% (w/v) (2-Hydroxypropyl)-*β*-cyclodextrin (Sigma Aldrich). Mice were monitored daily for weight loss and clinical signs. Investigators were not blind with respect to treatment.

### In vivo characterization of activity-based nanosensors

All activity-based nanosensor experiments were performed in accordance with institutional guidelines. Tumor-bearing mice and age-matched controls were administered activity-based nanosensor constructs via intratracheal intubation at 3.5, 5, 5.5, 6, and 7 weeks after tumor induction, with treatment with vehicle control or alectinib beginning at 5 weeks after tumor induction in Eml4-Alk mice and continuing for 2 weeks. Nanosensors for urinary experiments were synthesized by CPC Scientific (Sunnyvale, CA). The urinary reporter glutamate-fibrinopeptide B (Glu-Fib) was mass barcoded for detection by mass spectrometry. Sequences are provided in Table S1. Nanosensors were dosed (50 μL total volume, 20 μM each nanosensor) in mannitol buffer (0.28 M mannitol, 5 mM sodium phosphate monobasic, 15 mM sodium phosphate dibasic, pH 7.0–7.5) by intratracheal intubation. Anesthesia was induced by isoflurane inhalation, and mice were monitored during recovery. For intratracheal instillation, a volume of 50 μL was administered by passive inhalation following intratracheal intubation with a 22G flexible plastic catheter (Exel). Intratracheal instillation was immediately followed by a subcutaneous injection of PBS (200 μL) to increase urine production. Bladders were voided 60 min after nanosensor administration, and all urine produced 60–120 min after administration was collected using custom tubes in which the animals rest upon 96-well plates that capture urine. Urine was pooled and frozen at –80 °C until analysis by liquid chromatography tandem mass spectrometry (LC-MS/MS).

### LC-MS/MS reporter quantification

LC-MS/MS was performed by Syneos Health using a Sciex 6500 triple quadrupole instrument. Briefly, urine samples were treated with ultraviolet irradiation to photocleave the 3-Amino-3-(2-nitro-phenyl)propionic acid (ANP) linker and liberate the Glu-Fib reporter from residual peptide fragments. Samples were extracted by solid-phase extraction and analyzed by multiple reaction monitoring by LC-MS/MS to quantify concentration of each Glu-Fib mass variant. Analyte quantities were normalized to a spiked-in internal standard, and concentrations were calculated from a standard curve using peak area ratio (PAR) relative to the internal standard. Normalization to nanosensor stock concentrations and mean scaling were performed on PAR values to account for mouse-to-mouse differences in activity-based nanosensor inhalation efficiency and urine production.

### Statistical and machine learning analysis of urinary reporter data

Analyses of urinary reporter data were conducted using the analytic pipelines of the Protease Activity Analysis (PAA) package^52^, a publicly available Python package designed to process and visualize enzymatic activity datasets. For all urine experiments, PAR values were normalized to nanosensor stock concentrations and then mean-scaled across all reporters in a given urine sample prior to further statistical analysis. To identify differential urinary reporters, reporters were subjected to unpaired two-tailed *t* test followed by correction for multiple hypotheses using the Holm-Sidak method. *P*_*adj*_ < 0.05 was considered significant. For treatment response classification based on urinary reporter signatures, randomly assigned sets of paired data samples consisting of features (the mean scaled PAR values) and labels (the class membership; for example, Eml4-Alk or healthy) were used to train random forest classifiers with 100 trees. Estimates of out-of-bag error were used for cross-validation, and trained classifiers were tested on randomly assigned, held-out, independent test cohorts. Ten independent train-test trials were run for each classification problem, and classification performance was evaluated with receiver operating curve (ROC) statistics. Classifier performance was reported as the mean area under the curve (AUC) across the ten independent trials.

### AZP peptide synthesis and sequences

All AZPs were synthesized by CPC Scientific (Sunnyvale, CA) and reconstituted in dimethylformamide (DMF) unless otherwise specified. AZP sequences are provided in Table S3.

### In situ zymography with activatable zymography probes

To harvest lung tissue for AZP studies, mice were first euthanized by isoflurane overdose. Lungs were then filled with undiluted optimal-cutting-temperature (OCT) compound (Sakura) through catheterization of the trachea; the trachea was subsequently clamped; and lungs were extracted. Individual lobes were dissected and then immediately embedded and frozen in OCT compound. Cryosectioning was performed at the Koch Institute Histology Core. Prior to staining, slides were air dried, fixed in ice-cold acetone for 10 min, and then air dried. After hydration in PBS (3 × 5 min), tissue sections were blocked in protease assay buffer (50 mM Tris, 300 mM NaCl, 10 mM CaCl_2_, 2 mM ZnCl_2_, 0.02% (v/v) Brij-35, 1% (w/v) BSA, pH 7.5) for 30 min at room temperature. Blocking buffer was aspirated, and solution containing fluorescently labeled AZPs (1 μM each AZP) and a free poly-arginine control (polyR, 0.1 μM) diluted in the protease assay buffer was applied. Slides were incubated in a humidified chamber at 37 °C for 4 h. For inhibited controls, 400 μM AEBSF (Sigma Aldrich), 1 mM marimastat (Sigma Aldrich), or protease inhibitor cocktail (P8340, Sigma Aldrich) spiked with AEBSF and marimastat was added to the buffer at both the blocking and cleavage assay steps. For uninhibited conditions, dimethyl sulfoxide (DMSO) was added to the assay buffer to a final concentration of 3% (v/v). For co-staining experiments, primary antibodies (E-cadherin, AF748, R&D Systems, 4 μg/mL; vimentin, ab92547, Abcam, 0.5 μg/mL; CD31, AF3628, R&D Systems, 10 μg/mL; desmin, ab227651, Abcam, 1.32 μg/mL) were included in the AZP solution. Following AZP incubation, slides were washed in PBS (3 × 5 min), stained with Hoechst (Invitrogen, 5 μg/mL) and the appropriate secondary antibody if relevant (Invitrogen, 1:500), washed in PBS (3 × 5 min), and mounted with ProLong Diamond Antifade Mountant (Invitrogen). Slides were scanned on a Pannoramic 250 Flash III whole slide scanner (3DHistech).

### AZP precleavage characterization

The Z1 AZP (10 μM) was incubated with recombinant fibroblast activation protein (FAP) in FAP assay buffer (50 mM Tris, 1 M NaCl, pH 7.5) overnight at 37 °C to run the cleavage reaction to completion. After precleavage with recombinant FAP, the AZP solution was diluted to a final peptide concentration of 0.1 μM in protease assay buffer. Cognate intact Z1 AZP (1 μM) and precleaved Z1 AZP, each with a free polyR control (0.1 μM), were applied to fresh-frozen Eml4-Alk lung tissue sections (slide preparation described above) and incubated at 37 °C for 4 h. After AZP incubation, slides were washed, stained with Hoechst, mounted, and scanned.

### Immunohistochemistry and immunofluorescence staining

Lungs were excised and either embedded in OCT, as previously described, or fixed in 10% (v/v) formalin and embedded in paraffin. Prior to staining, slides with formalin-fixed, paraffin-embedded sections were subject to deparaffinization and antigen retrieval. Prior to staining, slides with fresh-frozen sections were air dried, fixed in ice-cold acetone for 10 min, air dried, and re-hydrated in PBS. Sections were stained with IgG isotype controls (ThermoFisher) and primary antibodies against vimentin (ab92547, Abcam, 1.0 μg/mL), E-cadherin (AF748, R&D Systems, 4.0 μg/mL), *α*-SMA (ab124964, Abcam, 1.5 μg/mL), CD31 (AF3628, R&D Systems, 10 μg/mL), VE-cadherin (36-1900, Invitrogen, 10 μg/mL), PDGFR*β* (3169, Cell Signaling, 1:100), and desmin (ab227651, Abcam, 1.32 μg/mL), as appropriate. For immunohistochemistry with *α*-SMA, slides were incubated with Rabbit-on-Rodent HRP-Polymer (RMR622, Biocare Medical) at native concentration for 30 min. For immunofluorescence, slides were washed in PBS, incubated with Hoechst (Invitrogen, 5 μg/mL) and the appropriate secondary antibody (Invitrogen, 1:500) for 30 min at room temperature, and washed in PBS. Slides were scanned as previously described.

### Quantification of AZP and immunofluorescence staining

AZP and immunofluorescence staining was quantified in QuPath 0.2.3^53^ and in ImageJ (NIH, v1.53). To perform cell-by-cell analysis, cell segmentation was performed using automated cell detection on the DAPI (nuclear) channel. For quantification of activity inhibition, AZP staining was calculated as a fold change of the mean nuclear AZP signal over the mean nuclear polyR signal. All nuclei within an individual tumor were averaged across that given tumor. Nuclei with a polyR intensity of less than 3 were excluded from analysis. For quantification of AZP intensity based on cell morphology and marker expression, cells were annotated as “vimentin-positive, spindle” if they were spindle-shaped and expressed vimentin; “E-cadherin-positive, cuboidal” if they were cuboidal-shaped and expressed E-cadherin; “vimentin-positive, round” if they were rounded and expressed vimentin. A random forest classifier was trained on all annotated cells (at least 20 cells per class) using multiple cellular features, including nuclear area and eccentricity, and mean cellular fluorescence intensity across all channels. The trained classifier was then applied to all cells across all tumors in the tissue section, and mean cellular fluorescence intensity was quantified. To assess the relationship between Z1 and CD31 staining, cell segmentation was performed as described above, and correlation was assessed between mean cellular Cy5 (Z1) intensity and mean cellular FITC (CD31) intensity. Density plots were generated using the dscatter function in MATLAB (R2019b). For quantification of co-localization, JACoP (Just Another Co-localization Plug-in)^54^ was used to determine pixel intensity-based correlations. Tumors were selected as regions of interest, and thresholds were chosen automatically using the Costes’ method. Co-localization was assessed via the pairwise correlation of pixel intensities within each tumor region of interest.

### In vivo administration of QZ1

QZ1 (Table S3) was reconstituted to 1 mg/mL in water, then reacted with mPEG-Maleimide, MW 2000 g/mol (Laysan Bio), for PEG coupling via maleimide-thiol chemistry. After completion of the reaction, the final compound was purified using high-performance liquid chromatography (HPLC). All reactions were monitored using HPLC connected with mass spectrometry. Characterization of the final compound, QZ1-(PEG2K), using HPLC and MALDI-MS indicated that products were obtained with more than 90% purity and at the expected molecular weight. Eml4-Alk mice (11–12 weeks post tumor induction) and age- and sex-matched C57BL/6J healthy controls (Jackson Labs; 18–22 weeks) were anesthetized using isoflurane inhalation (Zoetis). QZ1-(PEG2K) (4.5 nmoles in 0.9% NaCl) was administered intravenously via tail vein injection. Two hours after probe injection, mice were imaged on an in vivo imaging system (IVIS, PerkinElmer) by exciting Cy5 at 640 nm and measuring emission at 680 nm. Mice were subsequently euthanized by isoflurane overdose followed by cervical dislocation. Lungs were dissected and explanted for imaging via IVIS. Fluorescence signal intensity was quantified using the Living Image software (PerkinElmer, v4).

### Preparation of single-cell suspensions

Eml4-Alk mice (10–12 weeks post tumor induction) and age- and sex-matched C57BL/6J healthy controls (Jackson Labs; 18–22 weeks) were euthanized by isoflurane overdose, and lungs were excised, separated into lobes, and kept in a round cell culture dish (ThermoFisher) on ice. For tumor-bearing lungs, tumors were separated from healthy tissue using forceps and scissors under a dissecting microscope, and the dissected tumors and surrounding tissue were kept in 5 mL Eppendorf tubes (Sigma Aldrich) for preparation into single-cell suspension. Tissue was minced using Noyes spring scissors (Fine Science Tools) until pieces were less than 1 cm in size, with the visual appearance of ground meat. Minced tissue was then treated with digestion buffer, comprised of Hank’s Balanced Salt Solution (HBSS) without Ca^2+^, Mg^2+^ (ThermoFisher) with 2% (v/v) heat-inactivated fetal bovine serum (FBS), supplemented with DNase (Sigma Aldrich, 40 U/mL) and collagenase (Sigma Aldrich, 0.5 mg/mL). Samples were kept on ice during preparation and subsequently incubated at 37 °C for 30 min with end-over-end rotation. Samples were filtered using a 70 μm filter and diluted with RPMI-1640 (ThermoFisher) + 2% (v/v) heat-inactivated FBS. Cell suspension was centrifuged at 625 × *g* for 5 min, and the pellet was resuspended in ACK lysis buffer (ThermoFisher) for 2 min, followed by quenching with FACS buffer (PBS + 2% (v/v) heat-inactivated FBS). Cell suspension was centrifuged, and the supernatant was discarded.

For single cell RNA-seq, CD45+ cell depletion and viability enrichment was performed according to manufacturer’s instructions (StemCell Technologies). For depletion of CD45+ cells, the EasySep™ Mouse CD45 Positive Selection Kit (StemCell Technologies), together with a magnet for holding round-bottom or conical tubes (StemCell Technologies), was used for immunomagnetic positive selection of CD45+ leukocytes from the lung tissue preparation, with the goal of ultimately discarding isolated CD45+ cells. Briefly, target CD45+ cells were labeled with antibodies and magnetic particles, and then separated using the magnet. The supernatant suspension containing unlabeled (i.e., desired CD45- cells) was subsequently transferred into a fresh, clean tube. For viability enrichment, the EasySep™ Dead Cell Removal (Annexin V) Kit (StemCell Technologies), together with a magnet for holding round-bottom or conical tubes (StemCell Technologies), was used for column-free immunomagnetic depletion of apoptotic cells from the lung tissue preparation. Briefly, unwanted apoptotic cells were labeled with Annexin V, antibodies, and magnetic particles. Labeled cells were then magnetically separated from the remainder of the suspension, preserving desired cells that were subsequently transferred into a fresh, clean tube. Approximately 70% of cells from Eml4-Alk and healthy lungs were alive following viability enrichment. Following depletion of CD45+ cells and viability enrichment, FACS sorting was not performed prior to single cell RNA-seq.

### Activity-based cell sorting

Single cell lung suspensions from Eml4-Alk mice administered QZ1 were stained with the following antibodies (catalog number, vendor, clone, fluorophore, dilution): CD44 (563508, BD, IM7, BV605, 1:200), CD105 (564746, BD, MJ7/18, BV786, 1:200), Ly6-A/E (12-5981-81, ThermoFisher, D7, PE, 1:200), CD11b (557657, BD, M1/70, APC-Cy7, 1:200), CD45 (566439, BD, 30-F11, AF488, 1:400), and EpCAM (118216, BioLegend, G8.8, PE-Cy7, 1:200). Cells were stained for 20 min, and DAPI (1:10,000) was added immediately prior to sort. FACS sorting was performed on a FACSAria II (BD). Flow cytometry data was analyzed by the FlowJo software (Treestar). The sort strategy is shown in Fig. S21. At least 100,000 cells from each of the QZ1-hi and QZ1-lo compartments were collected into RPMI-1640 + 2% (v/v) heat-inactivated FBS and pelleted via centrifugation at 300 × *g* for 5 min. Cell pellets were lysed in Trizol (ThermoFisher), and RNA was extracted using RNEasy Mini Kits (Qiagen). Bulk RNA sequencing was performed by the MIT BioMicro Center. Libraries were prepared using the Clontech SMARTer Stranded Total RNAseq Kit (Clontech), precleaned, and sequenced using an Illumina NextSeq500 on an Illumina NextSeq flow cell. Feature counting was performed on BAM files using the Rsubread package in R (v4). Differential expression analysis on QZ1-hi vs QZ1-lo cells was performed using the DESeq2 package in R (v4). GSEA was performed using the clusterProfiler package and visualized using the enrichplot package in R (v4).

### Analysis of Eml4-Alk bulk RNA-seq dataset

Differential expression analysis over the entire transcriptome was performed on a bulk RNA-seq dataset from the Eml4-Alk mouse model of NSCLC, reported by Li et al.^26^, using the DESeq2 package in R (v4). The gene list was subsequently filtered to protease genes for visualization. The Li et al.^26^ dataset is publicly available with GEO accession GSE139349 https://www.ncbi.nlm.nih.gov/geo/query/acc.cgi?acc=GSE139349.

### RNA-seq of Eml4-Alk alveolar organoids

Alveolar type 2 (AT2) organoids were derived from *Trp*53^*fl*/*fl*^*Rosa*26^*LSL*−*Cas*9−2*A*−*eGFP*/+^ (*N* = 2) and *Trp*53^*fl*/*fl*^*Rosa*26^*LSL*−*tdTomato*/+^ (*N* = 1) mice. All source mice were females on a C57BL/6 background, with source *Rosa26* and *Trp53* strains acquired from Jackson Labs. Mice were between 8 and 15 weeks of age at the time of organoid derivation. Organoids were generated according to the protocol described in the work of Naranjo et al.^39^. These organoid lines were then infected with an adenovirus expressing Cre recombinase (Ad5-CMV-Cre) to generate *Trp53*-deficient, TdTomato-expressing (PT) and *Trp53*-deficient, Cas9-expressing (PC) organoids^39^. The Eml4-Alk (EA) inversion was induced in PT organoids using an adenovirus expressing sgRNAs targeting the EA inversion breakpoints and also expressing Cas9^25^. On the other hand, PC organoids were treated with a lentivirus expressing the same sgRNAs and Cre recombinase. PT Eml4-Alk (PTEA) and PC Eml4-Alk (PCEA) cultures were then incubated in media lacking FGF7, HGF, and NOGGIN to enrich for EA mutant cells. Whole RNA was then extracted from PTEA, PCEA, and two PC cultures (grown in full media) using phenol-chloroform extraction with TRIzol (Invitrogen), followed by purification with a RNAeasy MinElute Cleanup Kit (Qiagen). RNA purity was determined by UV-Vis spectrophotometry (NanoDrop) and all samples exhibited 260/280 ratios of greater than 1.98. Bulk RNA sequencing was performed by the MIT BioMicro Center. Libraries were sequenced using an Illumina NextSeq500 on an Illumina NextSeq flow cell with a read length of 75 nucleotides. Feature counting was performed on BAM files using the Rsubread package in R (v4). Differential expression analysis on Eml4-Alk-mutant vs. control PC organoids was performed using the DESeq2 package in R (v4). GSEA was performed using the clusterProfiler package and visualized using the enrichplot package in R (v4).

### Single cell RNA sequencing (scRNA-seq)

scRNA-seq was performed by the MIT BioMicro Center. Following preparation of the single-cell suspension after depletion of CD45+ cells and viability enrichment, single cells were processed using the 10X Genomics Single Cell 3ʹ platform using the Chromium Single Cell 3’ Library & Gel Bead Kit V2 kit (10X Genomics), per manufacturer’s protocol. Briefly, approximately 10,000 cells were loaded onto each channel and partitioned into Gel Beads in Emulsion (GEMs) in the 10x Chromium instrument. No FACS sorting was performed prior to loading on the 10x Chromium instrument. Following lysis of the captured cells, the released RNA was barcoded through reverse transcription in individual GEMs, and complementary DNA was generated and amplified. Libraries were constructed using a Single Cell 3’ Library and Gel Bead kit. The libraries were sequenced using an Illumina NovaSeq6000 sequencer on an Illumina NovaSeq SP flow cell with a read length of 100 nucleotides.

### Single cell RNA-seq data analysis

Raw gene expression matrices were generated for each sample by the Cell Ranger Pipeline (v.3.0.2) coupled with mouse reference version GRCm38. The output filtered gene expression matrices were analyzed by Python software (v.3.9.0) with the Scanpy package (v.1.7.2)^55^. The mean sequencing depth (mean number of raw reads per cell) was 9727 reads per cell for the Eml4-Alk lungs dataset and 15,929 reads per cell for the healthy lungs dataset. Genes expressed in at least three cells in the data and cells with >200 genes detected were selected for further analyses. Low quality cells were removed based on the number of total counts and the percentage of mitochondrial genes expressed. Specifically, cells with fewer than 4000 genes per cell (approximately <15,000 counts per cell) and less than 5% mitochondrial genes were retained, with thresholds selected based on the distribution of genes per cell vs. library size and the distribution of the percentage of counts in mitochondrial genes vs. library size. After removal of low quality cells, gene count matrices were total-count normalized, i.e. library-size normalized, to correct for library size, such that counts became comparable across cells. The gene counts for each cell were normalized by total counts over all genes with a scaling factor of 10,000, such that every cell had the same total of 10,000 after normalization. Normalized counts were log transformed (i.e., $\log (1+x)$ where *x* is the number of counts) to stabilize variance and facilitate comparison of relative differences in gene expression. The dataset was additionally filtered to remove cells expressing *Ptprc* (CD45). Features with high cell-to-cell variation were calculated. Principal component analysis (PCA) was conducted on highly variable genes using the scanpy.tl.pca function with default parameters on normalized and scaled data (Fig. S13). A k-nn neighborhood graph was computed over the PCA representation of the data, using the scanpy.pp.neighbors function with default parameters. The neighborhood graph was subsequently embedded via uniform manifold approximation and projection (UMAP) for dimensionality reduction, and cells were clustered in the UMAP embedding space using the Louvain algorithm with resolution 0.25. Cell types were annotated based on expression of known lung cell type marker genes (Table S4) curated from the literature^34,35^. All analyses and visualizations were implemented in Python with support from Scanpy^55^.

### Statistics and reproducibility

PCA and machine learning classification of activity-based nanosensor data was performed in Python (v.3.9.0) using the Protease Activity Analysis (PAA) package^52^. Differential gene expression analysis for bulk RNA-seq data was performed in R. scRNA-seq data analysis was performed in Python (v.3.9.0) using the Scanpy (v.1.7.2) package^55^. All remaining statistical analyses were conducted in Prism 9.0 (GraphPad). Sample sizes, statistical tests, and p-values are specified in figure legends.

Activity-based nanosensor, scRNA-seq, Eml4-Alk organoid, and activity-based cell sorting experiments were repeated twice with similar results. All other experiments (including AZP, immunohistochemistry, and immunofluorescence staining experiments) were repeated three times with similar results. Details on the reproducibility of representative images are provided in the relevant figure legends.

### Reporting summary

Further information on research design is available in the Nature Research Reporting Summary linked to this article.

## Supplementary information

Supplementary Information

Reporting Summary

## Data Availability

Source data to generate figures and tables are provided in publicly accessible repositories and in supplementary files. The Li et al.^26^ Eml4-Alk RNA-seq dataset is publicly available with GEO accession GSE139349 https://www.ncbi.nlm.nih.gov/geo/query/acc.cgi?acc=GSE139349. New RNA-seq and scRNA-seq data generated in this study are publicly available with GEO accession number GSE191079 https://www.ncbi.nlm.nih.gov/geo/query/acc.cgi?acc=GSE191079. The remaining data are available within the article as a supplementary source data file as well as on Zenodo under 10.5281/zenodo.6969494^56^. Source data are provided with this paper.

## Source data

Source Data

## Code availability

Code for analysis of data from in vivo activity-based nanosensors (Fig. 2) is available at https://github.com/avaamini/protease_activity_analysis, published as part of the Protease Activity Analysis (PAA) package^52,57^.
