# Supplementary material for: Multiscale profiling of protease activity in cancer
Source: Nat Commun. 2022 Oct 3;13:5745. doi: 10.1038/s41467-022-32988-5 (PMC9530178; doi:10.1038/s41467-022-32988-5)
Supplement: Supplementary file 1 — Supplementary Information [file 41467_2022_32988_MOESM1_ESM.pdf]

Supplementary Information for: Multiscale profiling of protease activity in cancer

Ava P. Amini[1-4†], Jesse D. Kirkpatrick[1,2†], Cathy S. Wang[1,5], Alex M. Jaeger[1], Susan Su[1,6], Santiago Naranjo[1,7], Qian Zhong[1], Christina M. Cabana[1,7], Tyler Jacks[1,7], Sangeeta N. Bhatia[1,2,8-12*]

[1]Koch Institute for Integrative Cancer Research, Massachusetts Institute of Technology, Cambridge, MA, USA

[2]Harvard MIT Division of Health Sciences and Technology, Massachusetts Institute of Technology, Cambridge, MA, USA

[3]Program in Biophysics, Harvard University, Boston, MA, USA

[4]Microsoft Research New England, Cambridge, MA, USA

[5]Department of Biological Engineering, Massachusetts Institute of Technology, Cambridge, MA, USA

[6]Department of Mechanical Engineering, Massachusetts Institute of Technology, Cambridge, MA, USA

[7]Department of Biology, Massachusetts Institute of Technology, Cambridge, MA, USA

[8]Department of Electrical Engineering and Computer Science, Massachusetts Institute of Technology, Cambridge, MA, USA

[9]Department of Medicine, Brigham and Women's Hospital, Boston, MA, USA

[10]Broad Institute of Massachusetts Institute of Technology and Harvard, Cambridge, MA, USA

[11]Wyss Institute at Harvard University, Boston, MA, USA

[12]Howard Hughes Medical Institute, Cambridge, MA, USA

[†]These authors contributed equally to this work.

[*]Corresponding author. E-mail: sbhatia@mit.edu.

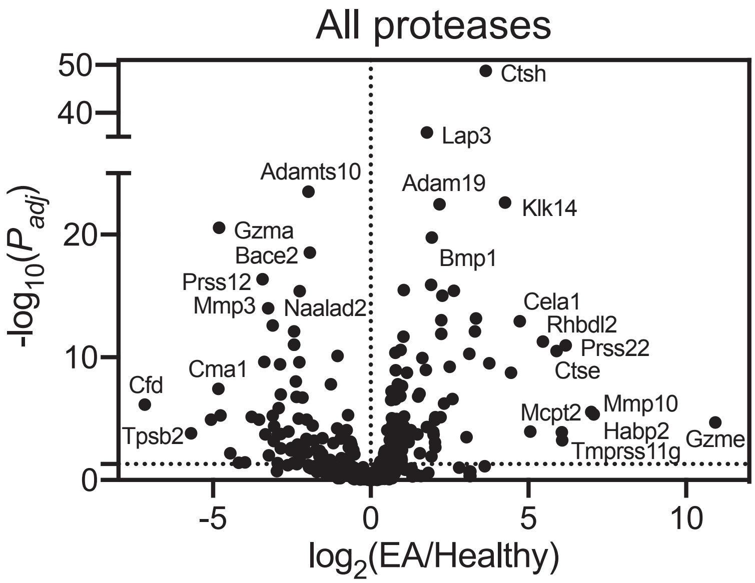

Figure S1: **Proteases are differentially expressed in the Eml4-Alk mouse model of NSCLC.** An existing bulk RNA-seq dataset from the Eml4-Alk model [26] was analyzed to identify endoproteases differentially expressed between Eml4-Alk mice (EA) and healthy controls (Healthy). Each point represents one protease gene. Significance was calculated by Wald test followed by adjustment for multiple hypotheses using the Benjamini-Hochberg correction. Dotted line is at $P_{adj} = 0.05$.

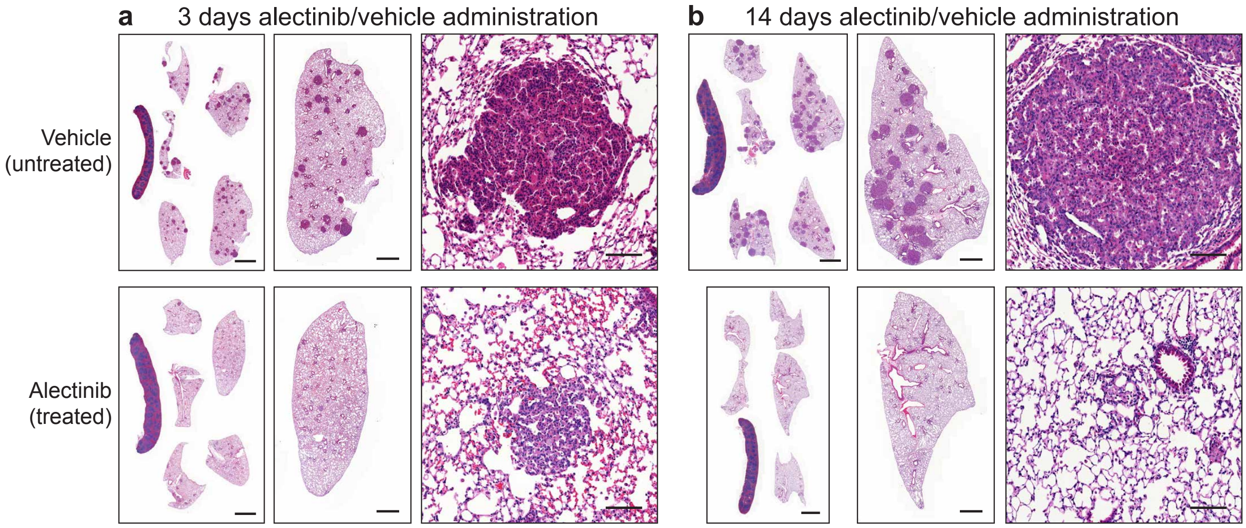

Figure S2: **Alectinib treatment induces tumor regression in the Eml4-Alk model. (a, b)** Images of representative lung sections and tumors (4 whole-lung sections per group; 5 lobes per lung set; 10-20 tumor regions per lobe) from vehicle control (untreated) and alectinib treated-mice, 3 days (a) and 14 days (b) post initiation of alectinib or vehicle administration. Scale bars = 2 mm, 1 mm, 100 μm for all lobes, single lobe, and individual tumors, respectively.

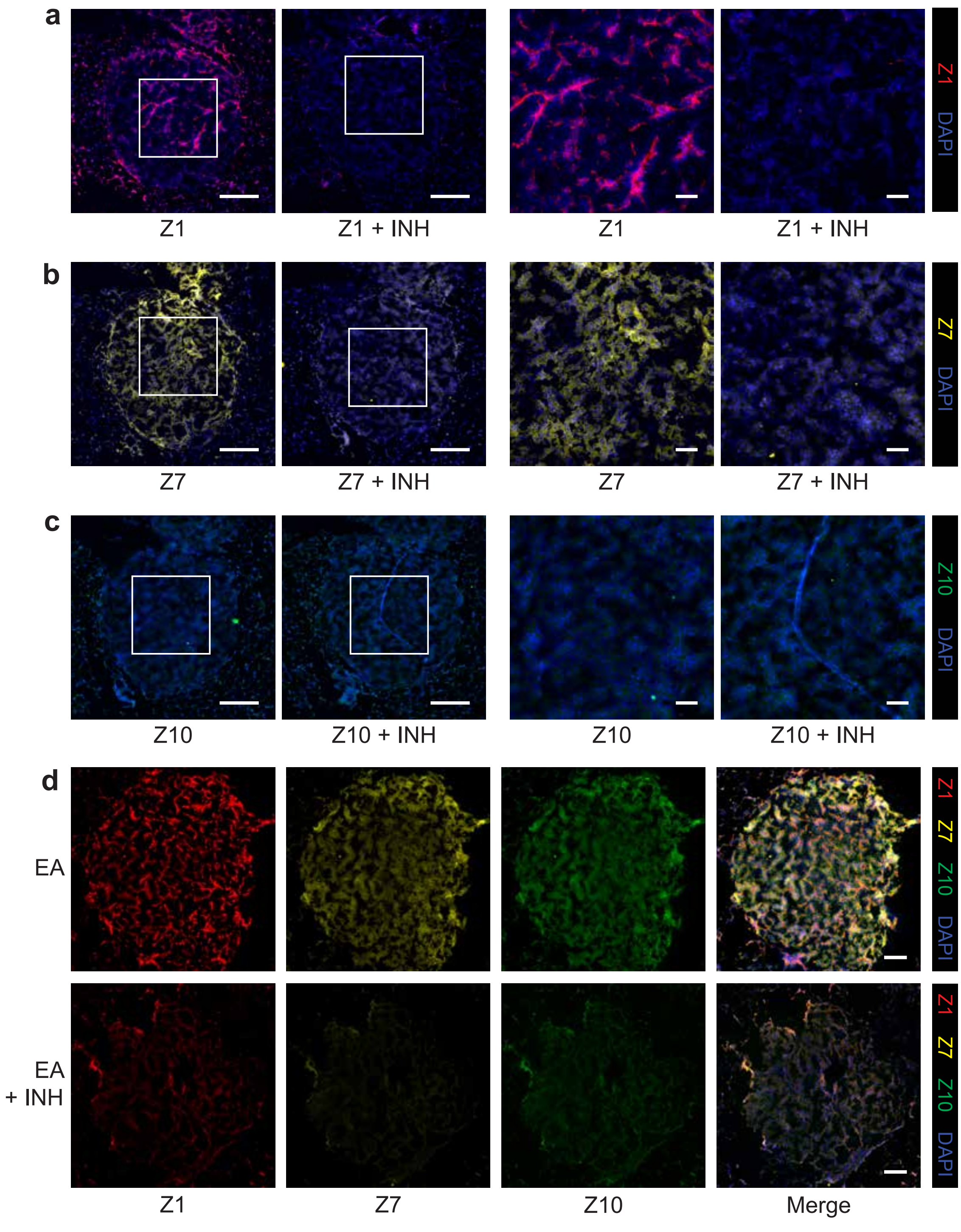

Figure S3: **AZPs nominated from *in vivo* profiling label Eml4-Alk tumors in a protease-dependent manner. (a-c)** Individual application of Z1 (a, red), Z7 (b, yellow), or Z10 (c, green), either alone or with broad-spectrum protease inhibitors (+ INH), to Eml4-Alk lung tissue sections. Sections were counterstained with DAPI (blue). Boxes show regions in higher magnification images on right. Scale bars = 200 μm, 50 μm (lower and higher magnification, respectively). Tumors shown are representative of all tumors in assayed tissue, with 7-10 tumors per lung tissue section. **(d)** Application of a multiplexed AZP cocktail of Z1 (red), Z7 (yellow), and Z10 (green), either uninhibited (EA) or with broad-spectrum protease inhibitors (EA + INH), to Eml4-Alk lung tissue sections. Scale bars = 100 μm.

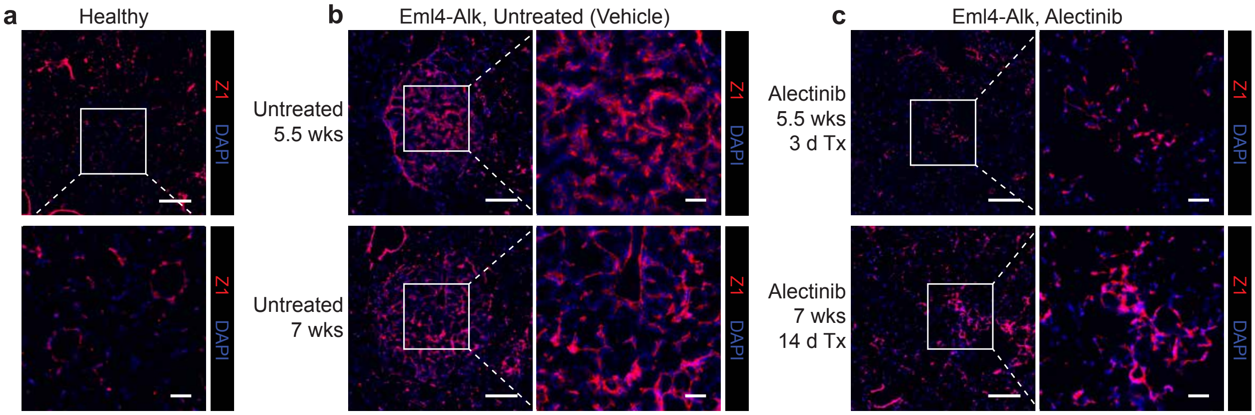

Figure S4: **Z1 labeling of Eml4-Alk tumors is abrogated by treatment with alectinib.** **(a)** Staining of representative section from healthy lungs with Z1 (red). **(b)** Staining of representative tumor regions from untreated Eml4-Alk mice at 5.5 (top) and 7 (bottom) weeks post tumor induction. **(c)** Staining of representative tumor regions from alectinib-treated Eml4-Alk mice at 5.5 (top) and 7 (bottom) weeks post tumor induction, corresponding to 3 (top) and 14 (bottom) days post initiation of alectinib treatment. All sections were counterstained with DAPI (blue). Scale bars = 200 μm, 50 μm (lower and higher magnification, respectively). Tumors shown are representative of all tumors in assayed tissue, with 7-10 tumors per lung tissue section.

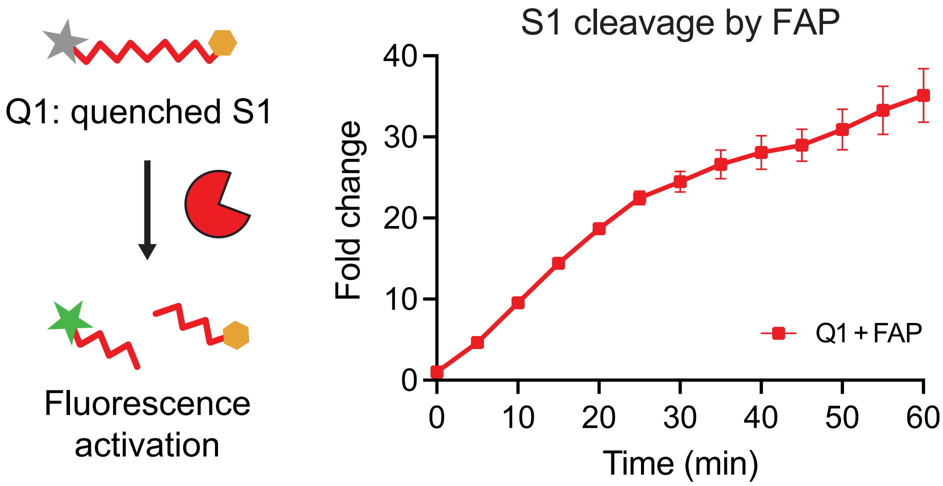

Figure S5: **Fibroblast activation protein (FAP) cleaves S1 *in vitro*.** The quenched probe Q1, incorporating the substrate S1, was incubated with recombinant FAP, and fluorescence activation was monitored over time (n = 2 replicates; mean ± s.d.).

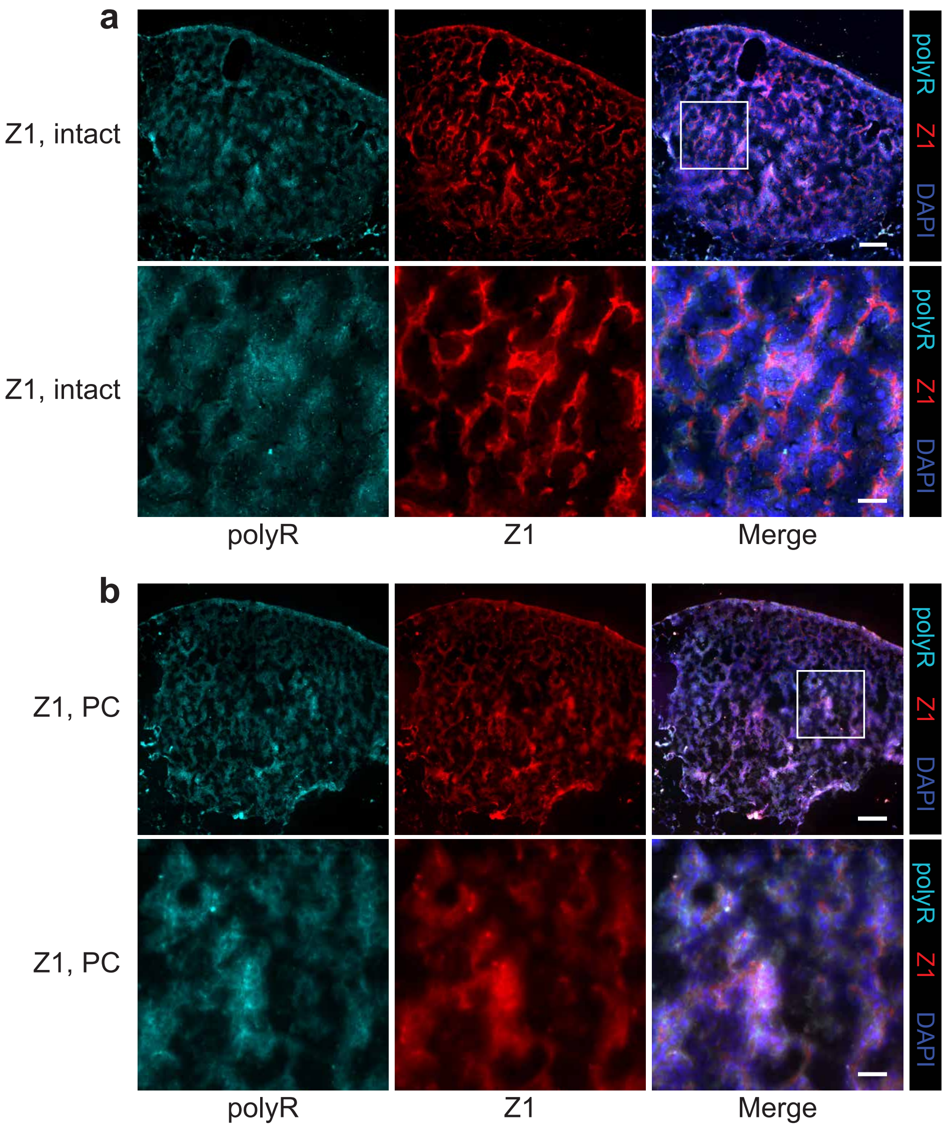

Figure S6: **Z1 localization pattern is not due to nonspecific binding. (a, b)** Tissue labeling and localization of intact (a) or FAP-precleaved Z1 (PC; b), together with free polyR control (cyan), within Eml4-Alk tumors. Probes were incubated at 37° C for 4 hours to allow cleavage of intact Z1 by endogenous, tissue-resident proteases. Scale bars = 100 μm, 25 μm (lower and higher magnification, respectively). Tumors shown are representative of all tumors in assayed tissue, with 7-10 tumors per lung tissue section.

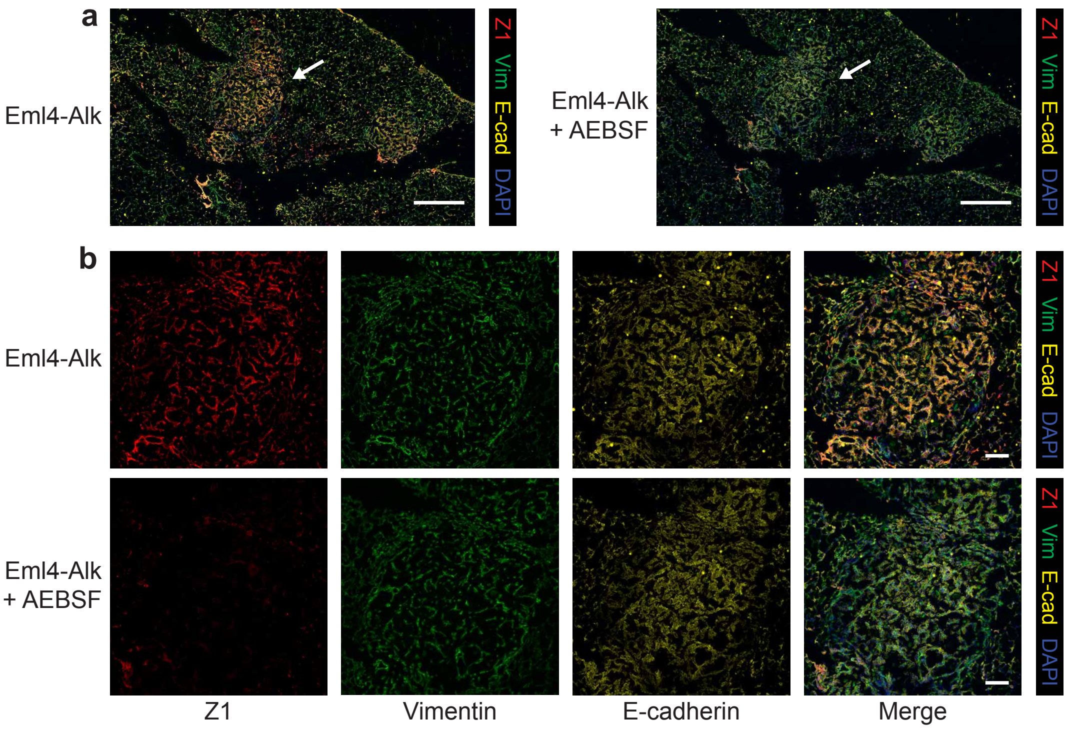

Figure S7: **Z1 staining is abrogated by serine protease inhibitor in tissues co-stained for vimentin and E-cadherin. (a)** Staining of Eml4-Alk lungs with Z1 (red), together with the mesenchymal marker vimentin (green) and the epithelial marker E-cadherin (yellow), with or without the serine protease inhibitor AEBSF. Scale bars = 500 μm. **(b)** Z1, vimentin, and E-cadherin staining in representative Eml4-Alk tumor regions, indicated by arrows in (a). Scale bars = 100 μm. Tumors shown are representative of all tumors in assayed tissue, with 7-10 tumors per lung tissue section.

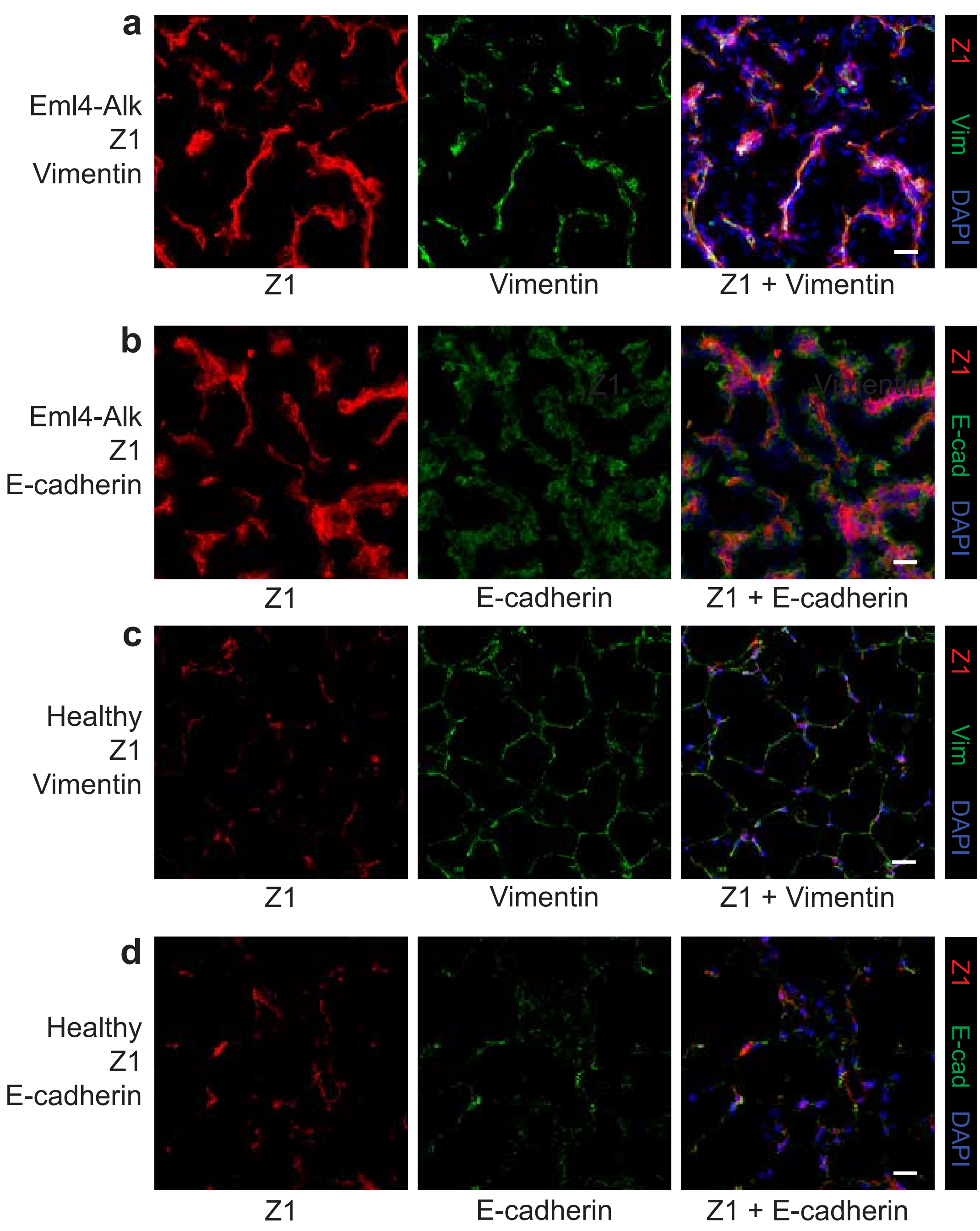

Figure S8: **Z1 localization pattern aligns closely with vimentin.** **(a)** Co-staining of representative Eml4-Alk tumor region with Z1 (red) together with vimentin (green). **(b)** Co-staining of representative Eml4-Alk tumor region with Z1 (red) together with E-cadherin (green). **(c)** Co-staining of healthy lungs with Z1 (red) together with vimentin (green). **(d)** Co-staining of healthy lungs with Z1 (red) together with E-cadherin (green). Scale bars = 25 μm. Tumors shown are representative of all tumors in assayed tissue, with 7-10 tumors per lung tissue section. Representative healthy regions are representative of four independent healthy lung sections assayed.

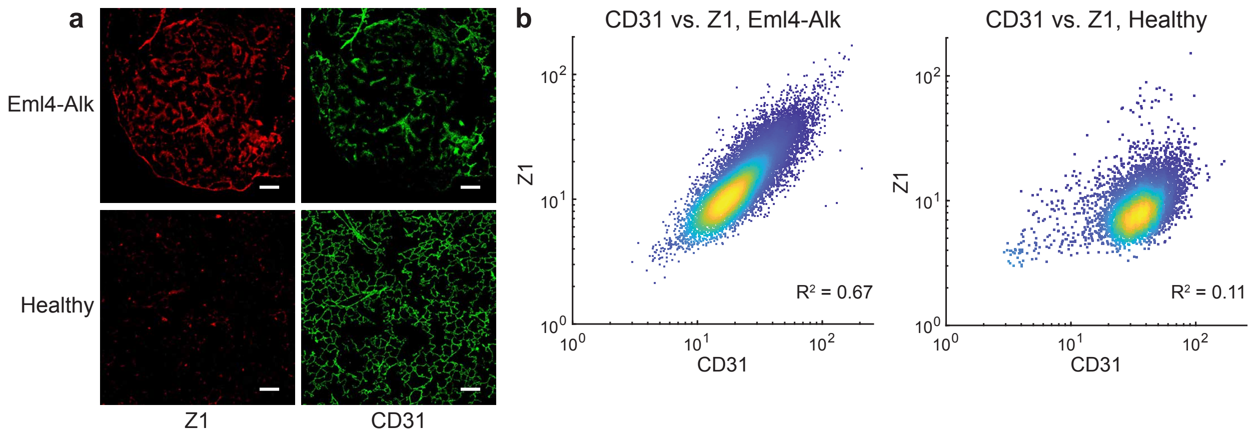

Figure S9: **Z1 staining correlates with CD31 staining in Eml4-Alk tumors.** **(a)** Application of Z1 (red) to lung tissue sections from Eml4-Alk and healthy control mice, together with the endothelial marker CD31 (green). Scale bars = 100 μm. Tumors shown are representative of all tumors in assayed tissue, with 7-10 tumors per lung tissue section. Representative healthy regions are representative of four independent healthy lung sections assayed. **(b)** Cell-by-cell quantification and correlation of Z1 and CD31 fluorescence intensity in Eml4-Alk (left) and healthy (right) lung tissue sections ($R^2 = 0.67$, $R^2 = 0.11$ for Eml4-Alk and healthy, respectively).

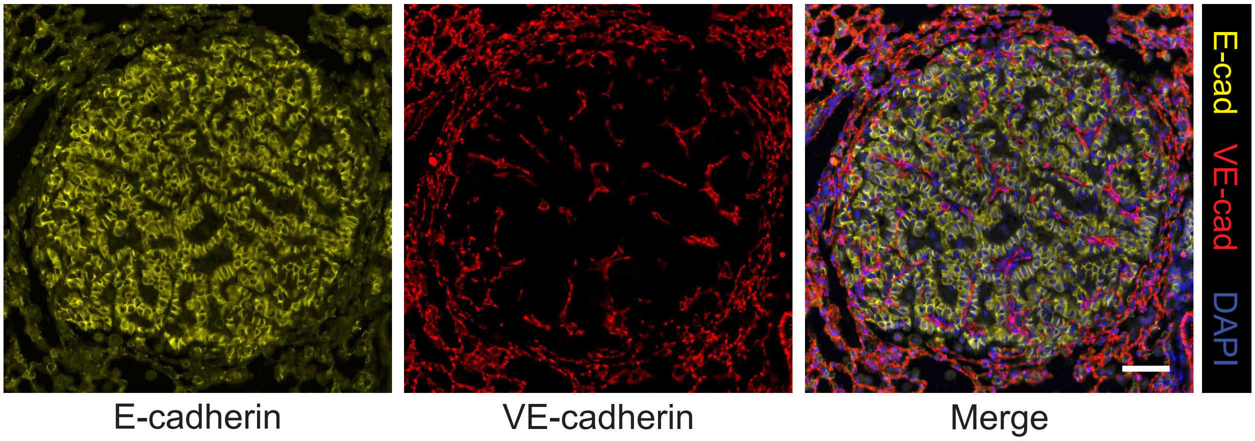

Figure S10: **Eml4-Alk tumors stain positively for E-cadherin and VE-cadherin.** Immunofluorescence staining for E-cadherin (yellow) and the endothelial marker VE-cadherin (red) in formalin-fixed, paraffin-embedded Eml4-Alk lung tissue sections, with images from a representative tumor shown. Sections were counterstained with DAPI (blue). Scale bar = 50 μm. Tumors shown are representative of all tumors in assayed tissue, with approximately 20-30 tumors per lung tissue section.

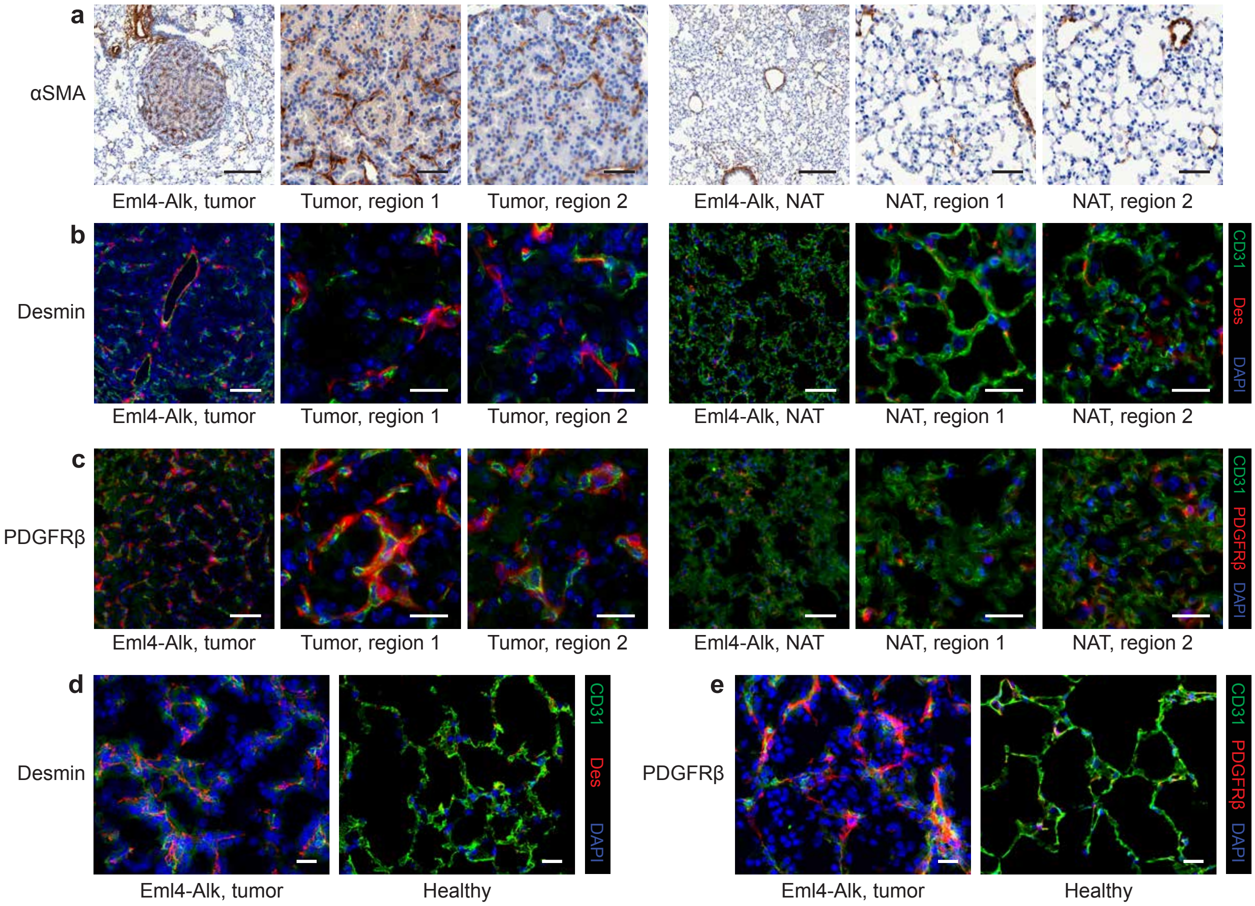

Figure S11: **Pericyte markers localize in close proximity to endothelial markers in Eml4-Alk tumors. (a)** Immunohistochemical staining for α-smooth muscle actin (αSMA) in representative Eml4-Alk tumor and normal adjacent tissue (NAT) regions. Scale bars = 200 μm (left columns per group), 50 μm (center and right columns per group). **(b, c)** Immunofluorescence staining for the endothelial cell marker CD31 (green) with each of the pericyte markers desmin (red, b) and PDGFRβ (red, c) in representative Eml4-Alk tumor and NAT regions. Sections were counterstained with DAPI (blue). Scale bars = 50 μm (left columns per group), 20 μm (center and right columns per group). **(d, e)** Immunofluorescence staining for the endothelial cell marker CD31 (green) with each of the pericyte markers desmin (red, d) and PDGFRβ (red, e) in representative Eml4-Alk tumor and healthy lung regions. Sections were counterstained with DAPI (blue). Scale bars = 20 μm. Tumors shown are representative of all tumors in assayed tissue, with 7-10 tumors per lung tissue section. Representative normal adjacent regions are representative across four independent Eml4-Alk lung sections assayed.

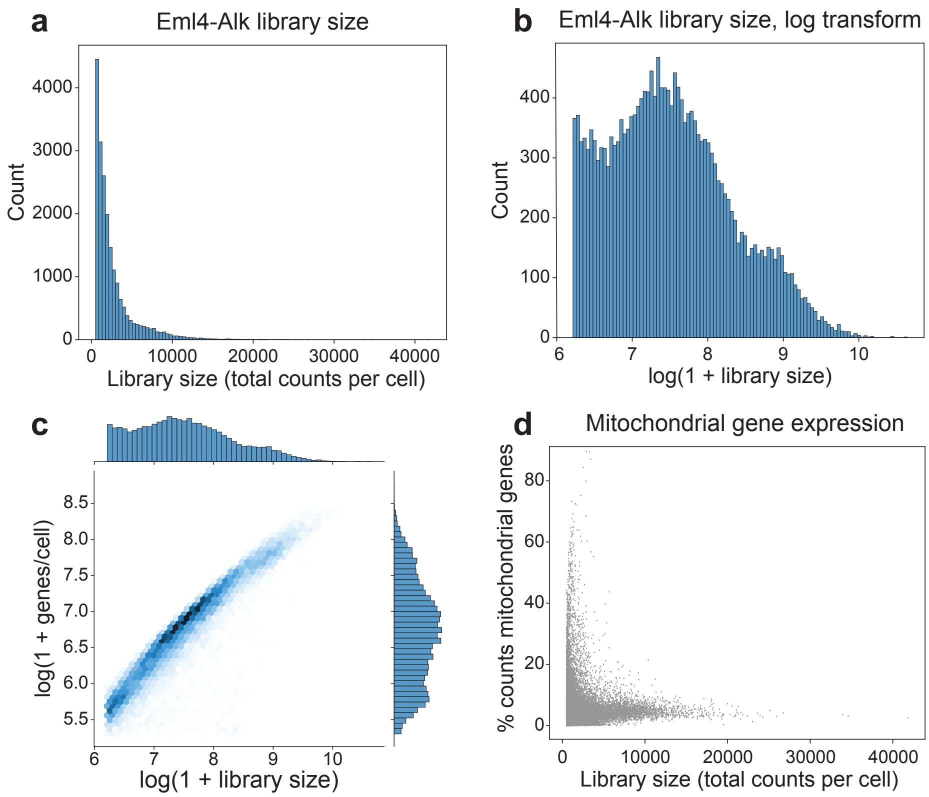

Figure S12: **Quality control metrics for scRNA-seq dataset of Eml4-Alk lungs.** **(a-b)** Distribution of library size (total counts per cell) prior to normalization, displayed as either total counts (a) or log transformed (b). **(c)** Distribution of the library size (total counts per cell) against the number of unique genes per cell, both log transformed. **(d)** Distribution of the library size (total counts per cell) against the distribution of mitochondrial gene expression per cell, expressed as a percentage. Metrics computed for a pooled sample of N = 3 mice; n_{EA} = 20,055 cells.

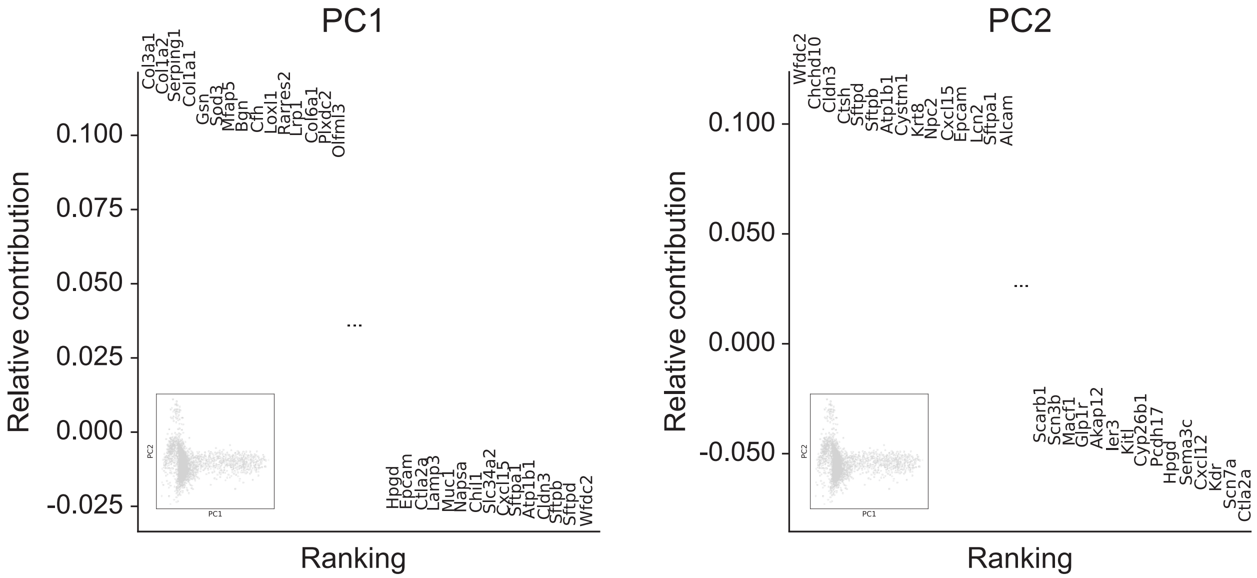

Figure S13: **Principal component gene rankings for Eml4-Alk lung scRNA-seq dataset.** Gene loadings and projection (inset) of the first two principal components for principal component analysis (PCA) of dataset of single cells from Eml4-Alk lungs (pooled sample of N = 3 mice; n_{EA} = 8,127 cells).

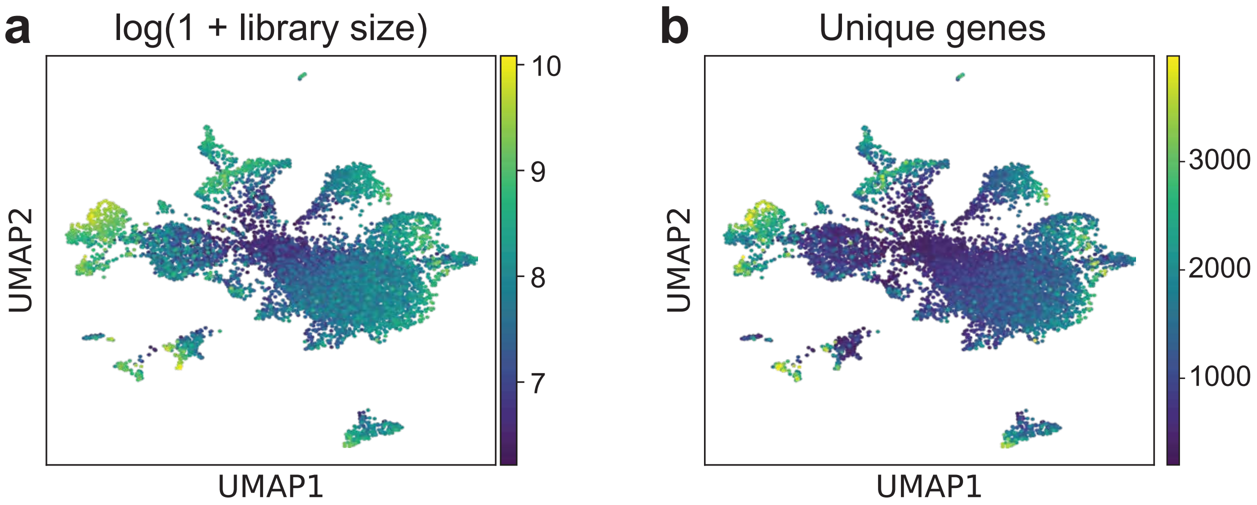

Figure S14: **Distributions of library size and number of genes expressed in Eml4-Alk scRNA-seq dataset. (a-b)** UMAP of dataset of single cells from Eml4-Alk lungs (pooled sample of N = 3 mice; n_{EA} = 8,127 cells), colored by library size (total counts, log-transformed; a) or number of unique genes (b). Individual dots correspond to individual cells.

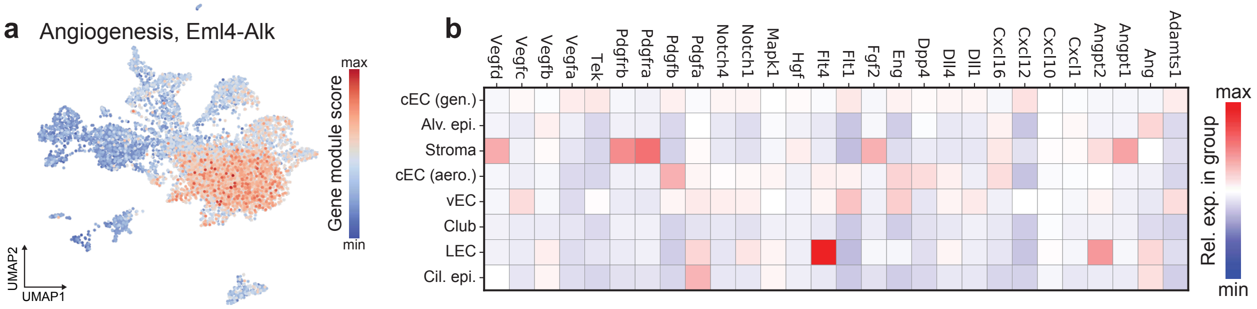

Figure S15: **Multiple cell populations in Eml4-Alk tumors express genes associated with angiogenesis. (a)** Collective expression score for angiogenesis gene module mapped onto UMAP of cells from Eml4-Alk lungs (pooled sample from N = 3 mice; n_{EA} = 8,127 cells). **(b)** Relative expression levels of individual genes in angiogenesis module, standardized across cell type clusters in Eml4-Alk lungs. Matrix plot is colored by mean expression values for a given gene, averaged across all cells in a cluster.

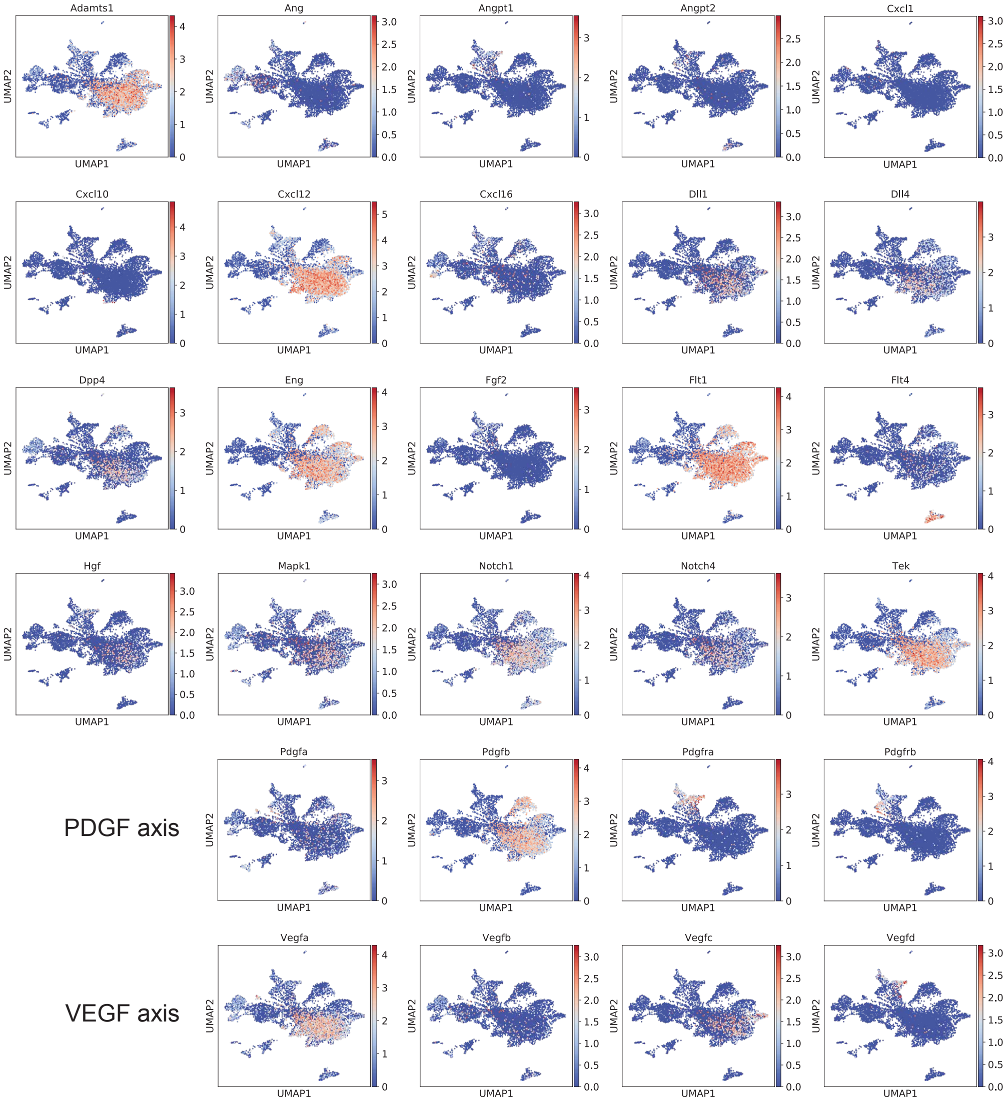

Figure S16: **Expression levels of angiogenesis module genes across the cellular landscape of Eml4-Alk lungs.** Expression of individual genes in the angiogenesis module (Fig. S15) was mapped onto the UMAP of cells from Eml4-Alk lungs (pooled sample from N = 3 mice; n_{EA} = 8,127 cells).

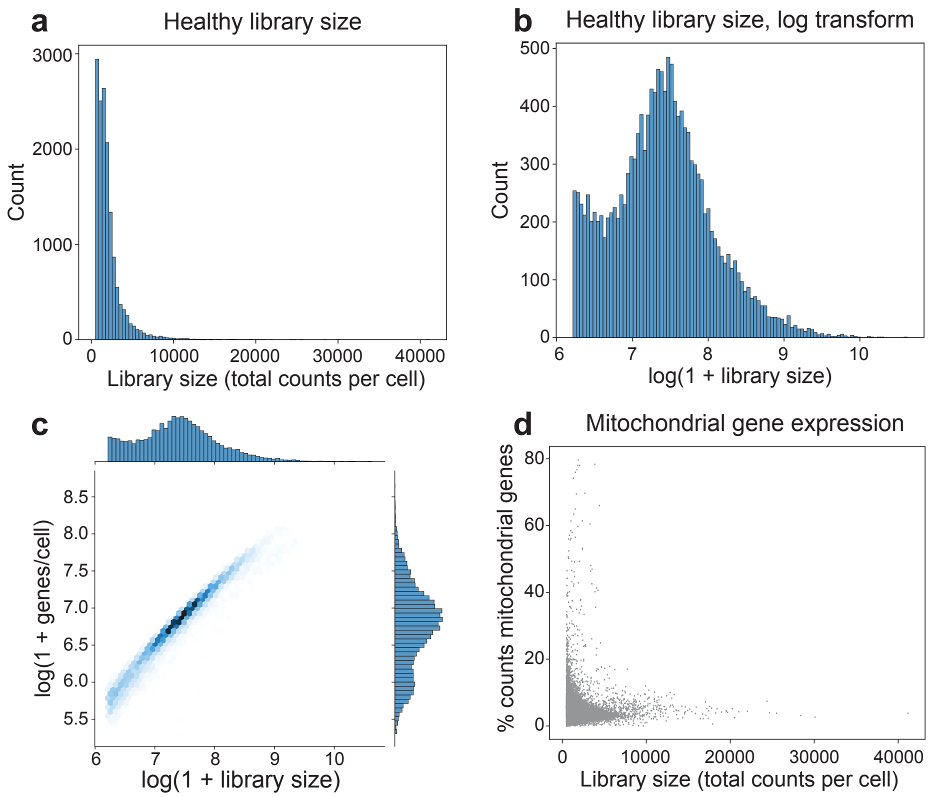

Figure S17: **Quality control metrics for scRNA-seq dataset of healthy lungs.** **(a,b)** Distribution of library size (total counts per cell) prior to normalization, displayed as either total counts (a) or log transformed (b). **(c)** Distribution of the library size (total counts per cell) against the number of unique genes per cell, both log transformed. **(d)** Distribution of the library size (total counts per cell) against the distribution of mitochondrial gene expression per cell, expressed as a percentage. Metrics computed for a pooled sample of N = 3 mice; n_H = 14,817 cells.

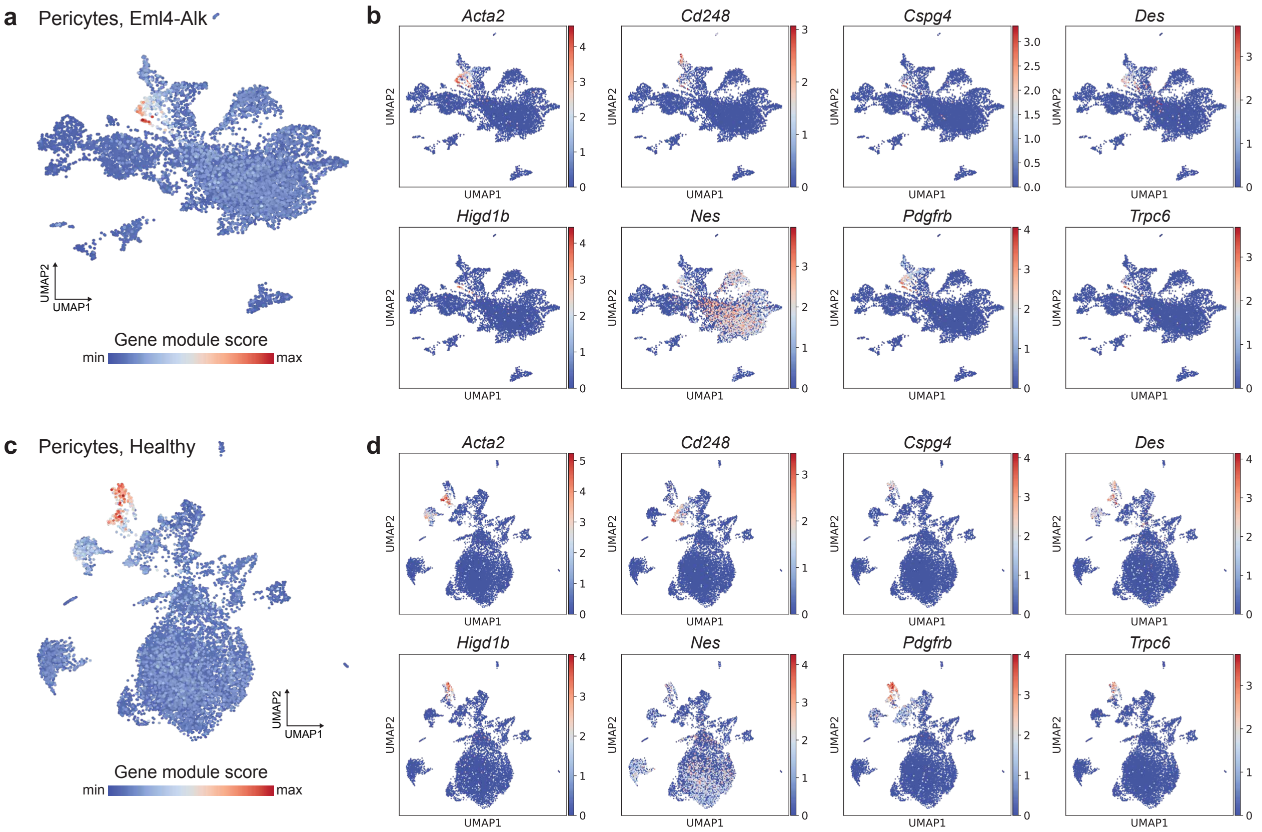

Figure S18: **Distribution of pericyte marker genes in Eml4-Alk and healthy lungs.** **(a)** Collective expression score for pericyte marker gene module mapped onto the UMAP of cells from Eml4-Alk lungs (pooled sample from N = 3 mice; n_{EA} = 8,127 cells). **(b)** Relative expression levels of individual pericyte marker genes mapped onto the UMAP of cells from Eml4-Alk lungs. **(c)** Collective expression score for pericyte marker gene module mapped onto the UMAP of cells from healthy lungs (pooled sample from N = 3 mice; n_H = 10,254 cells). **(d)** Relative expression levels of individual pericyte marker genes mapped onto the UMAP of cells from healthy lungs.

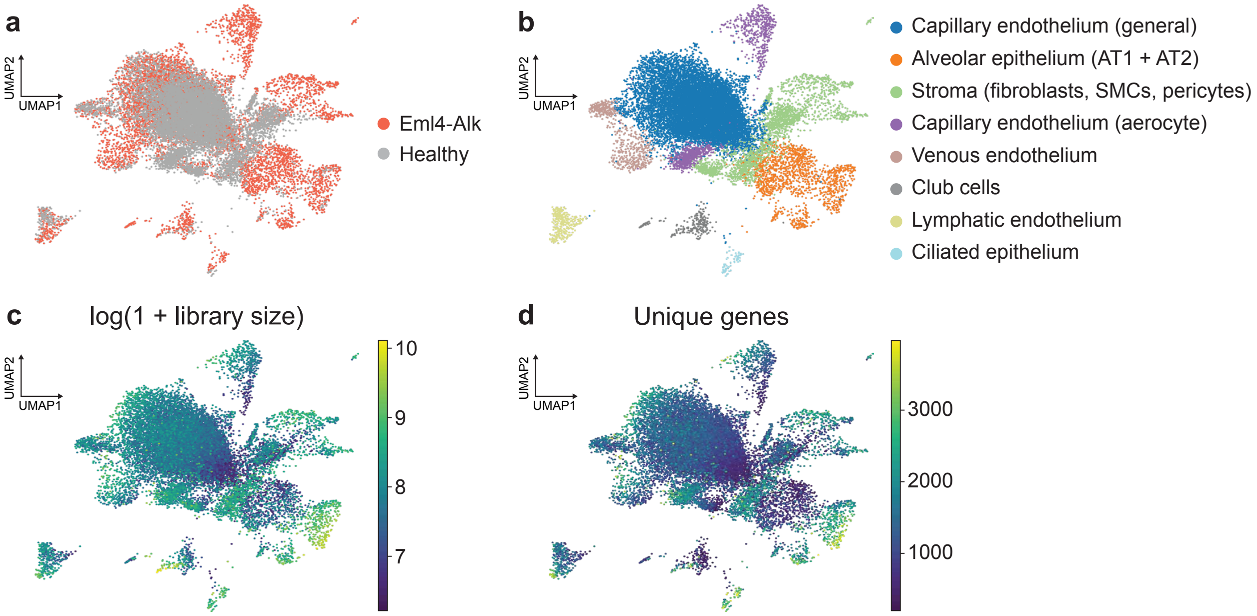

Figure S19: **Integrated comparison of single cell transcriptomic landscapes of Eml4-Alk and healthy lungs. (a-d)** UMAP of integrated dataset of single cells from Eml4-Alk and healthy lungs (pooled samples of N = 3 mice per condition; n = 18,381 cells total with n_{EA} = 8,127 and n_H = 10,254 cells from Eml4-Alk and healthy lungs, respectively). UMAP is colored by condition of origin (a), inferred cell type (b), library size (total counts, log-transformed; c), or number of unique genes (d). Individual dots correspond to individual cells.

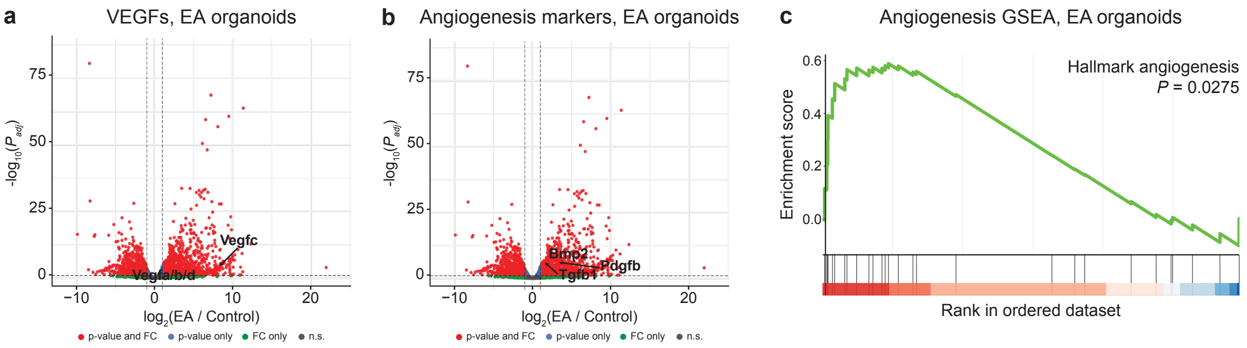

Figure S20: **Eml4-Alk-mutant alveolar organoids exhibit upregulation of genes associated with angiogenesis. (a,b)** Differential gene expression analysis, filtered for Vegf genes (a) and select angiogenesis markers (b), comparing the Eml4-Alk (EA) and control *p53-/-* alveolar organoid populations (n = 6 organoids per condition). Each point represents one gene. Significance was calculated by Wald test followed by adjustment for multiple hypotheses using the Benjamini-Hochberg correction. Dotted line is at $P_{adj} = 0.01$. **(c)** Gene set enrichment analysis (GSEA) plot showing enrichment of angiogenesis hallmark genes in Eml4-Alk organoids relative to control organoids (n = 6 organoids per condition).

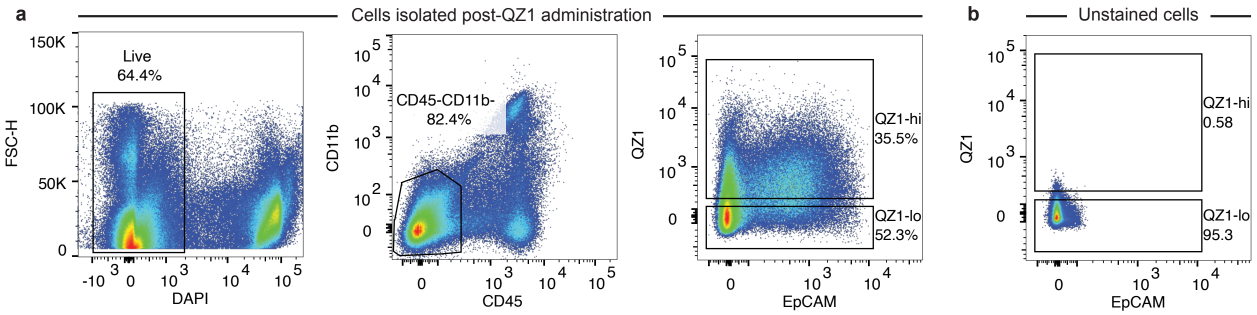

Figure S21: **Gating strategy for activity-based cell sorting.** **(a)** For activity-based cell sorting on the basis of QZ1 signal, cells were first sorted on the basis of viability by gating on DAPI signal. All non-hematopoietic cells were isolated by gating on CD45 negativity and CD11b negativity. Finally, cells were sorted for signal from the QZ1 activity probe into QZ1-high (QZ1-hi) and QZ1-low (QZ1-lo) populations by gating signal against EpCAM. **(b)** Gates for QZ1 signal, derived from distributions of QZ1 signal intensity in cells isolated following QZ1 administration (a), applied to unstained cells in lungs from mice not administered QZ1.

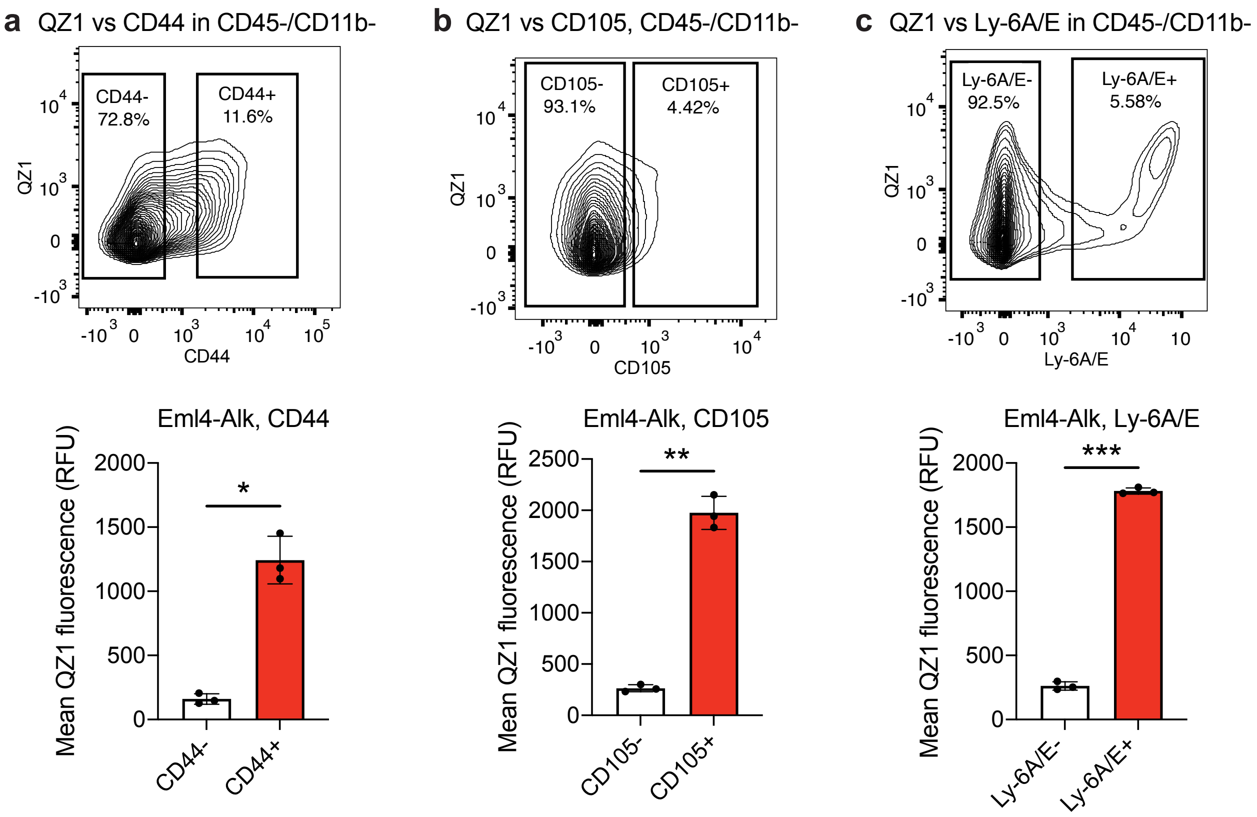

Figure S22: **QZ1 signal in Eml4-Alk tumors is increased in cell populations positive for mesenchymal markers. (a-c)** Flow cytometry plot (top) and quantification (bottom) of Cy5 (QZ1) fluorescence intensity in CD45-, CD11b- cells from Eml4-Alk lungs, gated by the cell surface markers CD44 (a), CD105 (b), and Ly-6A/E (c) (n = 3 biological replicates; mean ± s.d.; paired two-sided t-test, $*P = 0.0120$ for CD44, $**P = 0.00285$ for CD105, $***P = 0.000374$ for Ly-6A/E).

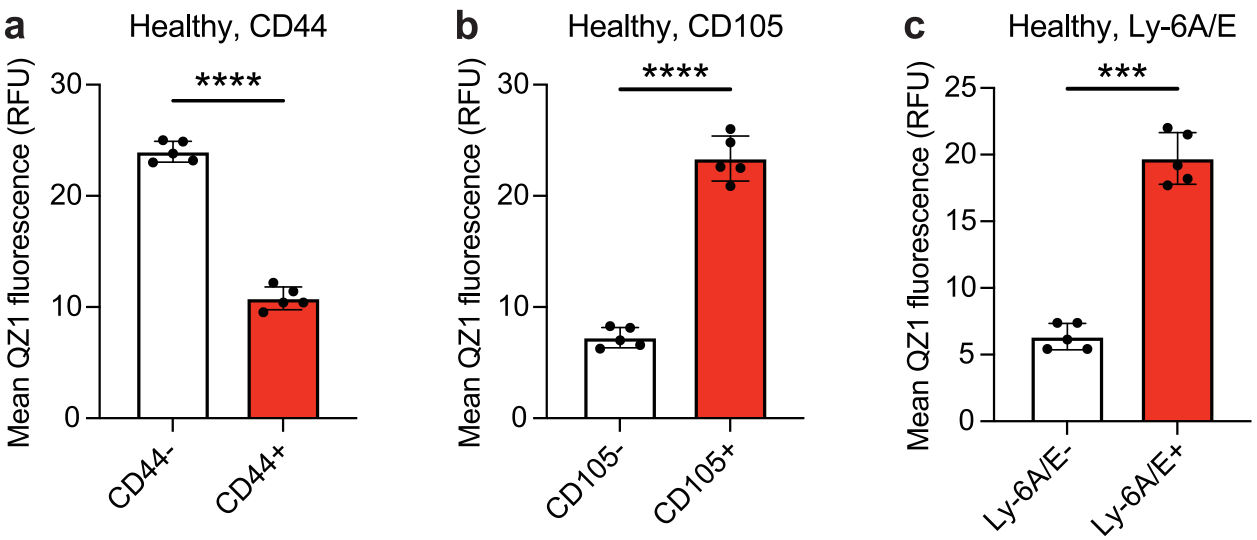

Figure S23: **Cells positive for mesenchymal markers in healthy lungs exhibit variable QZ1 labeling. (a-c)** Quantification of Cy5 (QZ1) fluorescence intensity in CD45-, CD11b- cells from healthy lungs, gated by the cell surface markers CD44 (a), CD105 (b), and Ly-6A/E (c) (n = 3 biological replicates; mean $\pm$ s.d.; paired two-sided t-test, $****P < 0.0001$ for CD44, $****P < 0.0001$ for CD105, $***P = 0.0002$ for Ly-6A/E.

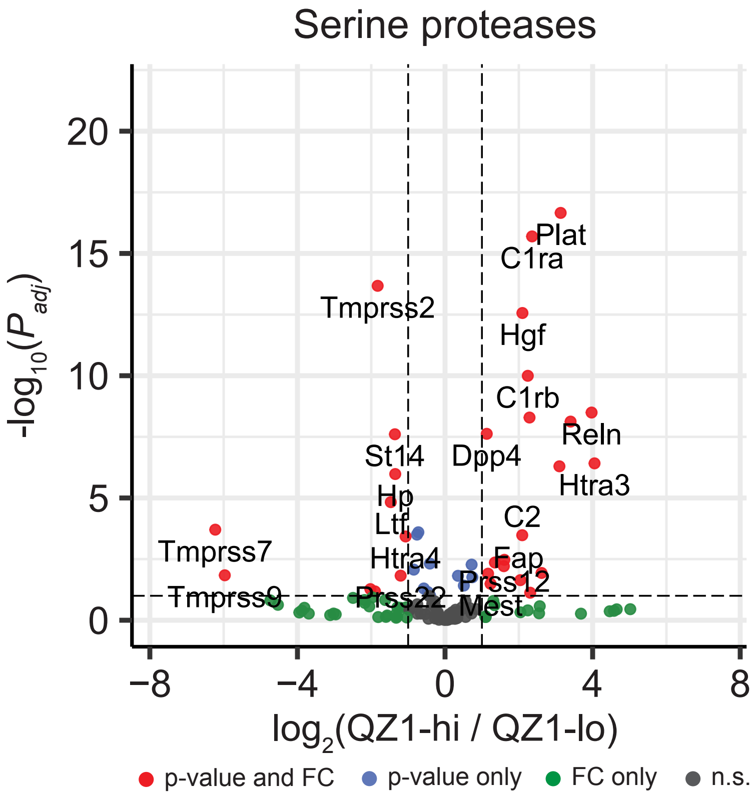

Figure S24: **Serine proteases are differentially expressed with respect to QZ1 labeling.** Differential gene expression analysis, filtered for serine proteases, comparing the QZ1-hi and QZ1-lo sorted populations from Eml4-Alk lungs. Each point represents one protease gene. Significance was calculated by Wald test followed by adjustment for multiple hypotheses using the Benjamini-Hochberg correction. Dotted line is at $P_{adj} = 0.01$.

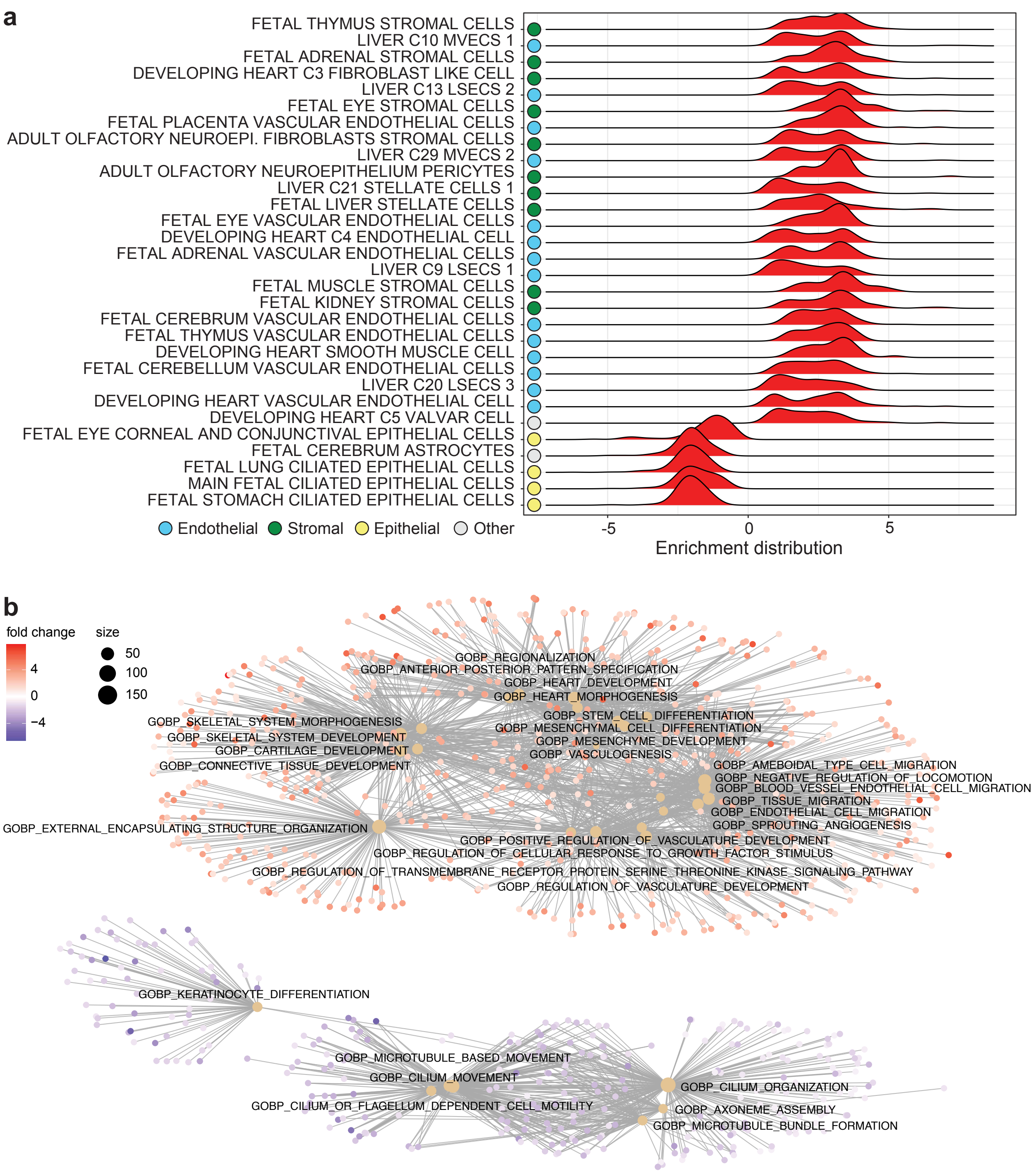

Figure S25: **QZ1-hi cells are enriched for gene sets associated with endothelial cells, stromal cells, and angiogenesis.** **(a)** Gene set enrichment analysis for cell type-specific expression modules based on a ranked list of differentially expressed genes from QZ1-hi and QZ1-lo populations isolated via activity-based cell sorting. **(b)** Enrichment map of functional gene ontology (GO) modules from Fig. 7G. Mutually overlapping gene sets are clustered together, while non-overlapping gene sets are distanced and clustered separately.

Name	Reporter	Photolabile group	Substrate	Nanocarrier
PP01	e(+2G)(+6V)ndneeGFFsAr	ANP	GGPQGIWGQC	PEG-8_{40kDa}
PP02	eG(+6V)ndneeGF(+1F)s(+1A)r	ANP	GGPVGLIGC	PEG-8_{40kDa}
PP03	e(+3G)(+1V)ndneeGFFs(+4A)r	ANP	GGPVPLSLVMC	PEG-8_{40kDa}
PP04	e(+2G)Vndnee(+2G)FFs(+4A)r	ANP	GGPLGLRSWC	PEG-8_{40kDa}
PP05	eGVndnee(+3G)(+1F)Fs(+4A)r	ANP	GGPLGVRGKC	PEG-8_{40kDa}
PP06	e(+2G)(+6V)ndnee(+3G)(+1F)(+1F)s(+1A)r	ANP	GGfPRSGGGC	PEG-8_{40kDa}
PP07	eG(+6V)ndnee(+3G)(+1F)Fs(+4A)r	ANP	GGLGPKGQTGC	PEG-8_{40kDa}
PP08	e(+3G)(+1V)ndneeG(+10F)FsAr	ANP	GGGSGRSANAKGC	PEG-8_{40kDa}
PP09	eGVndneeGF(+10F)s(+4A)r	ANP	GGKPISLISSGC	PEG-8_{40kDa}
PP10	e(+2G)(+6V)ndneeG(+10F)(+1F)s(+1A)r	ANP	GGILSRIVGGGC	PEG-8_{40kDa}
PP11	e(+3G)(+1V)ndnee(+2G)(+10F)Fs(+4A)r	ANP	GGSGSKIIGGGC	PEG-8_{40kDa}
PP12	eGVndneeG(+10F)(+10F)sAr	ANP	GGPLGMRGGC	PEG-8_{40kDa}
PP13	e(+2G)(+6V)ndnee(+3G)(+10F)(+1F)s(+4A)r	ANP	GGP-(Cha)-G-Cys(Me)-HAGC	PEG-8_{40kDa}
PP14	e(+3G)(+1V)ndnee(+2G)(+10F)(+10F)sAr	ANP	GGAPFEMSAGC	PEG-8_{40kDa}

Table S1: **Reporter and substrate sequences for *in vivo* activity-based nanosensors.** Lowercase letters: d-amino acids; ANP: 3-Amino-3-(2-nitro-phenyl)propionic acid; Cha: 3-Cyclohexylalanine; Cys(Me): methyl-cysteine

	**Eml4-Alk, Vehicle**	**Eml4-Alk, Alectinib**
3 days treatment (5.5 wks)	19	18
7 days treatment (6 wks)	13	12
14 days treatment (7 wks)	14	14
**Total**	**46**	**44**
Train	23	22
Test	23	22

Table S2: **Composition of cohorts for random forest classification.** Cohort numbers used to train and test random forest classifiers applied in Fig. 2g. Time points designate days post-treatment initiation and weeks post-tumor induction. Individual samples across the three time points were randomly selected for train or test cohorts per independent classification trial, according to a 50-50 split between train and test cohorts.

Name	Sequence	Readout
Z1	UeeeeeeeeXGGPQGIWGQGrrrrrrrrrX-k(Cy5)	Fluorescence (in situ)
Z7	UeeeeeeeeXGGLGPKGQTGGrrrrrrrrrX-k(Cy3)	Fluorescence (in situ)
Z10	UeeeeeeeeXGILSRIVGGGrrrrrrrrrX-k(FAM)	Fluorescence (in situ)
QZ1	(QSY21)-eeeeeeeee-c(PEG2K)-oGGPQGIWGQG-rrrrrrrrr-k(Cy5)	Fluorescence (in vitro / in vivo)
polyR	rrrrrrrrrX-k(Cy7)	Fluorescence (in situ)
S1	GGPQGIWGQC	Cleavage motif

Table S3: **Peptide sequences for activatable zymography probes.** Lowercase letters: d-amino acids; QSY21-Cy5: FRET pair, with Cy5 as fluorophore and QSY21 as quencher; PEG2K: (poly)ethylene-glycol, MW 2000 g/mol; o: 5-amino-3-oxopentanoyl; U: succinoyl; X: 6-aminohexanoyl

Annotated cell type label	Marker genes
Capillary endothelium (general)	*Cd36, Cd93, Gpihbp1, Plvap, Ptprb*
Capillary endothelium (aerocytes)	*Car4, Ednrb, Fibin, Prx*
Venous endothelium	*Amigo2, Slc6a2, Vegfc*
Lymphatic endothelium	*Mmrn1, Pdpn, Pecam1, Prox1, Thy1*
Alveolar epithelium: AT1 & AT2	*Annotated based on AT1 & AT2 marker genes*
Alveolar type 1	*Ager, Cdh1, Epcam, Hopx, Pdpn*
Alveolar type 2	*Aqp5, Cdh1, Epcam, Etv5, Muc1, Nkx2-1, Sftpb, Sftpc*
Club cells	*Cckar, Cdh1, Epcam, Scgb1a1, Scgb3a2*
Ciliated epithelium	*Ccdc78, Cdh1, Epcam, Fam183b, Foxj1*
Stroma	*Annotated based on below marker genes*
Pericyte	*Acta2, Cspg4, Des, Higd1b, Nes, Pdgfrb, Trpc6*
Adventitial fibroblast	*Entpd2, Pi16, Serpinf1*
Alveolar fibroblast	*Fgfr4, Slc7a10, Slc38a5*
Matrix fibroblast	*Col1a2, Col1a1, Fn1, Tcf21*
Myofibroblast	*Aspn, Fgf18, Pdgfra, Rgs2, Wif1*
Mesothelial	*Msln, Upk3b, Wt1*
Smooth muscle	*Acta2, Cnn1, Tagln*

Table S4: **Marker genes used to annotate cell types in scRNA-seq data from Eml4-Alk and healthy lungs.**

	**Cap. endothelium (general)**		**Stroma**		**Cap. endothelium (aerocyte)**		**Ciliated epithelium**	
**Gene**	$\log_2$(EA/WT)	P_{adj}	$\log_2$(EA/WT)	P_{adj}	$\log_2$(EA/WT)	P_{adj}	$\log_2$(EA/WT)	P_{adj}
Pdgfa	0.578	8.29×10^{-1}	0.07840	1.00	1.670	3.85×10^{-1}	0.999	1.00
Pdgfb	0.676	1.39×10^{-25}	0.824	1.00	0.835	1.77×10^{-11}	10.331	1.00
Pdgfra	-0.396	1.00	-0.0295	1.00	-0.145	1.00	11.335	1.00
Pdgfrb	-1.776	1.00	0.178	1.00	-7.183	1.00	0.000	1.00
Cxcl12	1.453	1.73×10^{-195}	1.542	5.77×10^{-19}	1.537	1.32×10^{-2}	11.335	1.00

Table S5: **PDGF signaling genes are differentially expressed in scRNA-seq data from Eml4-Alk and healthy lungs.** Differential gene expression analysis was conducted on scRNA-seq data from cells within each of the indicated cell compartments, and results were filtered for genes from the PDGF signaling axis. Significance was calculated by the Wilcoxon rank-sum two-sided test with Benjamini-Hochberg correction.
