# Supplementary material for: Multiscale profiling of protease activity in cancer
Source: Nat Commun. 2022 Oct 3;13:5745. doi: 10.1038/s41467-022-32988-5 (PMC9530178; doi:10.1038/s41467-022-32988-5)
Supplement: Supplementary file 2 — Reporting Summary [file 41467_2022_32988_MOESM2_ESM.pdf]

nature portfolio

Corresponding author(s): Sangeeta N. Bhatia

Last updated by author(s): Aug 7, 2022

Reporting Summary

Nature Portfolio wishes to improve the reproducibility of the work that we publish. This form provides structure for consistency and transparency in reporting. For further information on Nature Portfolio policies, see our Editorial Policies and the Editorial Policy Checklist.

Statistics

For all statistical analyses, confirm that the following items are present in the figure legend, table legend, main text, or Methods section.

n/a	Confirmed	
☐	☒	The exact sample size (n) for each experimental group/condition, given as a discrete number and unit of measurement
☐	☒	A statement on whether measurements were taken from distinct samples or whether the same sample was measured repeatedly
☐	☒	The statistical test(s) used AND whether they are one- or two-sided<br>*Only common tests should be described solely by name; describe more complex techniques in the Methods section.*
☐	☒	A description of all covariates tested
☐	☒	A description of any assumptions or corrections, such as tests of normality and adjustment for multiple comparisons
☐	☒	A full description of the statistical parameters including central tendency (e.g. means) or other basic estimates (e.g. regression coefficient) AND variation (e.g. standard deviation) or associated estimates of uncertainty (e.g. confidence intervals)
☐	☒	For null hypothesis testing, the test statistic (e.g. F, t, r) with confidence intervals, effect sizes, degrees of freedom and P value noted<br>*Give P values as exact values whenever suitable.*
☒	☐	For Bayesian analysis, information on the choice of priors and Markov chain Monte Carlo settings
☒	☐	For hierarchical and complex designs, identification of the appropriate level for tests and full reporting of outcomes
☐	☒	Estimates of effect sizes (e.g. Cohen's d, Pearson's r), indicating how they were calculated

Our web collection on statistics for biologists contains articles on many of the points above.

Software and code

Policy information about availability of computer code

Data collection: Activatable zymography probe and immunofluorescence staining was quantified in QuPath (Bankhead 2017, v.0.2.3) and in ImageJ (NIH, v1.53). Signal for IVIS imaging was collected using the Living Image software (PerkinElmer, v4). Flow cytometry data was quantified in FlowJo (BD, v10).

Data analysis: Activatable zymography probe and immunofluorescence staining was quantified in QuPath (Bankhead 2017, v.0.2.3) and in ImageJ (NIH, v1.53). For quantification of co-localization, JACoP (Just Another Co-localization Plug-in) in ImageJ (NIH, v1.53) was used to determine pixel intensity-based correlations.

Fluorescence signal intensity for IVIS images was quantified using the Living Image software (PerkinElmer, v4). Flow cytometry data was quantified in FlowJo (BD, v10), and staining density plots were generated using the dscatter function in MATLAB (R2019b).

Analysis of bulk RNA-seq data was performed in R (v4). Specifically, feature counting was performed on BAM files using the Rsubread package. Differential expression analysis was performed using the DESeq2 package in R (v4). GSEA was performed using the clusterProfiler package and visualized using the enrichplot package.

All analyses and machine learning classification of activity-based nanosensor data was performed in Python (v.3.9.0) using the protease activity analysis package, published in Soleimany et al., ACS Omega 2022, and available at https://github.com/avaamini/protease_activity_analysis. scRNA-seq data analysis was performed in Python (v.3.9.0) using the scanpy (v.1.7.2) package.

All remaining statistical analyses were conducted in Prism 9.0 (GraphPad). Sample sizes, statistical tests, and p-values are specified in figure

legends.

For manuscripts utilizing custom algorithms or software that are central to the research but not yet described in published literature, software must be made available to editors and reviewers. We strongly encourage code deposition in a community repository (e.g. GitHub). See the Nature Portfolio guidelines for submitting code & software for further information.

Data

Policy information about availability of data

All manuscripts must include a data availability statement. This statement should provide the following information, where applicable:

- Accession codes, unique identifiers, or web links for publicly available datasets
- A description of any restrictions on data availability
- For clinical datasets or third party data, please ensure that the statement adheres to our policy

Source data to generate figures and tables are provided in publicly accessible repositories and in supplementary files. The Li et al. Eml4-Alk RNA-seq dataset is available with GEO accession GSE139349 (https://www.ncbi.nlm.nih.gov/geo/query/acc.cgi?acc=GSE139349). New RNA-seq and scRNA-seq data generated in this study are publicly available with GEO accession number GSE191079 (https://www.ncbi.nlm.nih.gov/geo/query/acc.cgi?acc=GSE191079). The remaining data are available within the article as a supplementary source data file as well as on Zenodo under DOI 10.5281/zenodo.6969494 (https://doi.org/10.5281/zenodo.6969494).

Human research participants

Policy information about studies involving human research participants and Sex and Gender in Research.

Reporting on sex and gender	N/A
Population characteristics	N/A
Recruitment	N/A
Ethics oversight	N/A

Note that full information on the approval of the study protocol must also be provided in the manuscript.

Field-specific reporting

Please select the one below that is the best fit for your research. If you are not sure, read the appropriate sections before making your selection.

☒ Life sciences ☐ Behavioural & social sciences ☐ Ecological, evolutionary & environmental sciences

For a reference copy of the document with all sections, see nature.com/documents/nr-reporting-summary-flat.pdf

Life sciences study design

All studies must disclose on these points even when the disclosure is negative.

Sample size	Sample size is indicated in the figure legend for each experiment. No sample-size calculations were performed. Sample sizes were chosen based on results from prior work and preliminary experiments. Namely, for in vivo activity-based nanosensor experiments, sample sizes were chosen based on the prior works of Kirkpatrick et al., Science Translational Medicine 2020, and Anahtar et al., PNAS 2022; for in situ activatable zymography probe and immunofluorescence experiments, sample sizes were chosen based on the prior work of Soleimany et al., Cancer Research 2021. Preliminary experiments were also used to choose sample sizes. Sample size was determined to be adequate based on the magnitude and consistency of measurable differences between groups.
Data exclusions	No data was excluded for both in vitro and in vivo experiments.
Replication	Activity-based nanosensor, scRNA-seq, Eml4-Alk organoid, and activity-based cell sorting experiments were repeated twice with similar results. All other experiments (including AZP, immunohistochemistry, and immunofluorescence staining experiments) were repeated three times with similar results. Details on the reproducibility of representative images are provided in the relevant figure legends.
Randomization	For in vivo experiments, groups were established upon tumor induction. All mice analyzed were sex- and age-matched.
Blinding	Investigators were not blinded to the groups and treatments during experiments. Due to the longitudinal nature of the in vivo experiments and the administration of treatments longitudinally, investigators were required to know the identity and treatment cohort for individual mice throughout the experiment. For in vitro and in situ experiments, these studies were conducted with knowledge of the conditions and samples being assayed, as is standard in the life sciences and required by the nature of the assay. Data reported for all experiments are not subjective but based on the quantitative assays described.

Reporting for specific materials, systems and methods

We require information from authors about some types of materials, experimental systems and methods used in many studies. Here, indicate whether each material, system or method listed is relevant to your study. If you are not sure if a list item applies to your research, read the appropriate section before selecting a response.

Materials & experimental systems

n/a	Involved in the study	
☐	☒	Antibodies
☒	☐	Eukaryotic cell lines
☒	☐	Palaeontology and archaeology
☐	☒	Animals and other organisms
☒	☐	Clinical data
☒	☐	Dual use research of concern

Methods

n/a	Involved in the study	
☒	☐	ChIP-seq
☐	☒	Flow cytometry
☒	☐	MRI-based neuroimaging

Antibodies

Antibodies used	For immunofluorescence and immunohistochemistry, IgG isotype controls (ThermoFisher) were used at the relevant concentrations. Primary antibodies, vimentin (ab92547, Abcam, 1.0 ug/mL), E-cadherin (AF748, R&D Systems, 4.0 ug/mL), alpha-SMA (ab124964, Abcam, 1.5 ug/mL), CD31 (AF3628, R&D Systems, 10 ug/mL), VE-cadherin (36-1900, Invitrogen, 10 ug/mL), PDGFRbeta (3169, Cell Signaling, 1:100), and desmin (ab227651, Abcam, 1.32 ug/mL). For immunofluorescence, species-appropriate secondary antibodies (Invitrogen) were used at 1:500 dilution. For immunohistochemistry, Rabbit-on-Rodent HRP-Polymer (RMR622, Biocare Medical) was used at native concentration.<br><br>For flow cytometry, single cell lung suspensions from Eml4-Alk mice administered QZ1 were stained with the following antibodies (catalog number, vendor, clone, fluorophore, dilution): CD44 (563508, BD, IM7, BV605, 1:200), CD105 (564746, BD, MJ7/18, BV786, 1:200), Ly6-A/E (12-5981-81, ThermoFisher, D7, PE, 1:200), CD11b (557657, BD, M1/70, APC-Cy7, 1:200), CD45 (566439, BD, 30-F11, AF488, 1:400), and EpCAM (118216, BioLegend, G8.8, PE-Cy7, 1:200).
Validation	Antibodies were validated via comparison to IgG isotype controls or compensation controls for IF/IHC and flow cytometry experiments, respectively. This involved staining consecutive sections or replicate samples, respectively, with IgG isotype or compensation controls, respectively. In comparison to these controls, antibodies were validated based on inspection of signal relative to signal isotype or compensation controls, following confirmation that isotype or compensation controls did not exhibit real signal. Further validation information was provided by the manufacturer, namely through knockout validation, Western blot validation, and IF/IHC validation on samples with known target expression.

Animals and other research organisms

Policy information about studies involving animals; ARRIVE guidelines recommended for reporting animal research, and Sex and Gender in Research

Laboratory animals	Female C57BL/6J mice (Jackson Labs) were used for all studies. Tumors were initiated in 6-10 week old female C57BL/6J mice by chromosomal rearrangement of the Eml4 and Alk genes, induced by intratracheal administration of 50 uL adenovirus expressing the Ad-EA vector (VQAd Cas9 ALK EML4 072415; Viraquest). These mice are referred to as `Eml4-Alk' mice in the manuscript. Animals were monitored daily throughout all studies. Healthy control cohorts consisted of age- and sex-matched mice, female C57BL/6J from Jackson Labs, that did not undergo intratracheal administration of Ad-EA adenovirus.<br><br>Mice were housed in an MIT on-premise animal facility with a 12/12 light-dark cycle, temperature range of 70 +/- 2 degrees Fahrenheit, and humidity range of of 30-70%.
Wild animals	The study did not involve wild animals.
Reporting on sex	Female C57BL/6J mice (Jackson Labs) were used for all studies.
Field-collected samples	The study did not involve field-collected samples.
Ethics oversight	All animal studies were approved by the Massachusetts Institute of Technology (MIT) committee on animal care (MIT protocol 0420-023-23) in accordance with institutional and national policies. Reporting was in compliance with Animal Research: Reporting In Vivo Experiments (ARRIVE) guidelines.

Note that full information on the approval of the study protocol must also be provided in the manuscript.

Flow Cytometry

Plots

Confirm that:

- [x] The axis labels state the marker and fluorochrome used (e.g. CD4-FITC).
- [x] The axis scales are clearly visible. Include numbers along axes only for bottom left plot of group (a 'group' is an analysis of identical markers).
- [x] All plots are contour plots with outliers or pseudocolor plots.
- [x] A numerical value for number of cells or percentage (with statistics) is provided.

Methodology

Sample preparation	Eml4-Alk mice (10–12 weeks post tumor induction) and age- and sex-matched C57BL/6J healthy controls (Jackson Labs; 18–22 weeks) were euthanized by isoflurane overdose, and lungs were excised, separated into lobes, and kept in a round cell culture dish (ThermoFisher) on ice. For tumor-bearing lungs, tumors were separated from healthy tissue using forceps and scissors under a dissecting microscope, and the dissected tumors and surrounding tissue were kept in 5 mL Eppendorf tubes (Sigma Aldrich) for preparation into single-cell suspension. Tissue was minced using Noyes spring scissors (Fine Science Tools) until pieces were less than 1 cm in size, with the visual appearance of ground meat. Minced tissue was then treated with digestion buffer, comprised of Hank's Balanced Salt Solution (HBSS) without Ca2+, Mg2+ (ThermoFisher) with 2% (v/v) heat-inactivated fetal bovine serum (FBS), supplemented with DNase (40 U/mL, Sigma Aldrich) and collagenase (0.5 mg/mL, Sigma Aldrich). Samples were kept on ice during preparation, and subsequently incubated at 37C for 30 minutes with end-over-end rotation. Samples were filtered using a 70 um filter and diluted with RPMI-1640 (ThermoFisher) + 2% heat-inactivated FBS. Cell suspension was centrifuged at 625 g for 5 minutes and the pellet was resuspended in ACK lysis buffer (ThermoFisher) for 2 minutes, followed by quenching with FACS buffer (PBS + 2% (v/v) heat-inactivated FBS). Cell suspension was centrifuged and supernatant was discarded.
Instrument	FACSAria II (BD)
Software	FlowJo (BD, v10)
Cell population abundance	For each population (QZ1-hi and QZ1-lo), approximately 100,000 cells were collected. The purity of the fractions were not directly assessed, as our approach was agnostic to the specific cell populations within each fraction. Downstream analysis with bulk RNA sequencing revealed that the QZ1-hi population was enriched for endothelial cells and pericytes, while the QZ1-lo population was enriched for epithelial cells.
Gating strategy	The complete gating strategy is included in the manuscript supplement (Figure S21). FSC-H vs DAPI was used to gate on all live cells (DAPI < 2x10^3 RFU). CD11b vs CD45 was then used to isolate all non-hematopoietic cells (gated on CD45 < 300 RFU and CD11b < ~200 RFU). Finally, QZ1 (Cy5) was used to sort QZ1-hi vs QZ-lo cells. QZ1-hi cells had Cy5 fluorescence > ~300 RFU, while QZ1-lo cells had Cy5 fluorescence < ~100 RFU.

- [x] Tick this box to confirm that a figure exemplifying the gating strategy is provided in the Supplementary Information.
